# DNA Sequential Logic Circuits for Reversible Counters and Dynamic Biomolecular Sensing

**DOI:** 10.1002/advs.202505793

**Published:** 2025-06-19

**Authors:** Tianci Xie, Changjiang Li, Minghao Hu, Xingyu Zhong, Junbin Xiao, Zhen Zhang, Ze Wang, Tongbo Wu

**Affiliations:** ^1^ Orthopedics Department Wuhan Children's Hospital (Wuhan Maternal and Child Healthcare Hospital) Tongji Medical College Huazhong University of Science & Technology Wuhan 430015 China; ^2^ School of Pharmacy Tongji Medical College Huazhong University of Science and Technology Wuhan 430030 China; ^3^ GI Cancer Research Institute Tongji Hospital Tongji Medical College Huazhong University of Science and Technology Wuhan 430030 China; ^4^ Department and Institute of Urology Tongji Hospital Tongji Medical College Huazhong University of Science and Technology Wuhan 430030 China; ^5^ The First Affiliated Hospital and College of Clinical Medicine Henan University of Science and Technology Luoyang 471003 China

**Keywords:** biological information storage, biosensors, DNA structures, latch, sequential logic circuit

## Abstract

The capacity to retain and precisely release historical data at the right moments is typically managed by sequential logic circuits within computer systems. However, the reusability and autonomy of DNA sequential logic circuits still need to be developed. To bridge this gap, a series of sequential logic circuits are implemented by constructing a DNA strand replacement system regulated by a nicking enzyme (nickase). This nickase‐integrated system dynamically resolves the thermodynamic‐kinetic conflict, offering spatiotemporal control over strand displacement. These circuits include Set‐Reset latches (SR‐latches) constructed with NOR and NAND gates, along with Data latches (D‐latches), are designed with simplicity, autonomy, and reusability. Furthermore, addition, subtraction, and reversible counters leveraging these foundational circuits are successfully constructed. These latches are further applied to transient miRNA recording, environmental toxin detection, and real‐time ATP imaging in living cells.

## Graphical abstract

In this work, a DNA sequential logic circuit is constructed that combines reusability and autonomy for the first time, including SR (Set‐Reset)‐latches composed of NOR gates, SR‐latches composed of NAND gates, and D (Data)‐latches. After fully utilizing the characteristics of each latch, to counter, toxin and miRNA detection and intracellular dynamic ATP monitoring are successfully applied.

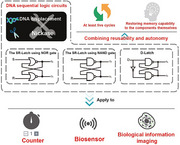

## 1 Introduction

The elegance of life arises from the extensive molecular‐level computation and information processing. As the primary medium for information processing in living organisms, DNA has proven to be highly versatile, specific, and self‐assembled. It is an ideal biomaterial for constructing various basic logic gates and advanced logic devices. Numerous studies have exploited these properties to develop DNA‐based devices that exhibit superior adaptability and facile conformational changes.^[^ ^1^, ^2^, ^3^, ^4^, ^5^, ^6^, ^7^ ^]^ In comparison to silicon‐based electronics, DNA‐based devices possess enhanced biocompatibility, thereby holding great potential for applications in environmental monitoring, drug delivery, and intelligent medical diagnostics.^[^ ^8^, ^9^, ^10^, ^11^, ^12^, ^13^, ^14^, ^15^, ^16^ ^]^

Logic circuits are formed by interconnecting multiple logic gates to achieve specific functions. Digital circuits can be categorized into two types based on their logic functions: combinational logic circuits and sequential logic circuits.^[^ ^17^, ^18^, ^19^ ^]^ Combinational logic circuits operate with continuous inputs; once the input is terminated, the information is lost (Figure S1A, Supporting Information). To address this limitation, sequential circuits are designed to store information while performing functions and release it when necessary (Figure S1B, Supporting Information).^[^ ^17^, ^18^, ^19^ ^]^ While combinational logic circuits solely perform computational functions,^[^ ^1^, ^2^, ^3^ ^]^ advanced computers, similar to humans relying on past experiences, require memory to make present decisions. These computers can not only compute but also store data and determine the subsequent output by combining stored information with present input. Sequential logic circuits are essential for performing these functions.

In contrast to combinational logic circuits, constructing DNA sequential logic circuits entails addressing two crucial issues. First, restoring memory capability to the components themselves, ensuring that their sequential responsiveness is independent of human manipulation and that uniform inputs are processed each time. Second, the components should be reusable, allowing them to store information and release it to determine the following output. Some existing molecular sequential circuit elements utilize the absorbance changes of supramolecular dyes in various environments to achieve functions such as keyboard lock and counting.^[^ ^20^, ^21^, ^22^, ^23^ ^]^ However, these elements are limited in functionality and lack expandability. Moreover, their response information predominantly pertains to ions, making them less suitable for the dynamic living environment. Most current DNA sequential circuits solely enable ordered reactions without storing or releasing information, these circuits resemble cascaded chemical reactions more than sequential logic circuits.^[^ ^24^, ^25^, ^26^, ^27^, ^28^ ^]^

The latch serves as the fundamental component of a sequential logic circuit,^[^ ^17^, ^18^, ^19^ ^]^ and our objective is to construct an autonomous sequential circuit element starting from the latch. Latches are digital circuits designed to store a single bit of information, maintaining its value until refreshed by new input signals. They are employed in digital systems as temporary storage elements for binary information.^[^ ^17^, ^18^, ^19^ ^]^ Two main types of latches exist: SR (Set‐Reset)‐latches and D (Data)‐latches (Figure S2, Supporting Information). SR‐latches represent the simplest form, comprising two inputs: S (Set) and R (Reset). In the SR‐latch, the latch's state before the input's action can be stored as present state Q. The S input 1 sets the secondary state Q* to 1, while the R input 1 resets Q* to 0. For an SR‐latch built from NOR gates, S = R = 1 is a forbidden state, while S = R = 0 is the holding state. For an SR‐latch built from NAND gates, S = R = 1 is the holding state, while S = R = 0 is the forbidden state. (Figure S2A–D, Supporting Information). To circumvent such forbidden states, the D‐latch replaces S and R with a single input (D) coupled with a NOT gate and two AND gates. The NOT gate on the input makes sure the S and the R inputs are always opposites, to avoid the invalid state of both being 1 or 0. The two AND gates create a new input, E (Enable), which controls when the latch responds to the signal at the D input. This means that the output Q can only change when the enable signal is 1. In simple terms if E = 0 then the latch is as good as non‐existent (Figure S2E,F, Supporting Information). So, in the DNA circuit, the presence of the D‐latch defaults to E = 1, so we can omit the E input and simplify the D‐latch to that shown in and Figure S2G,H (Supporting Information).

In this study, we adopt the design concept of a “simple single structure to achieve complex functions”.^[^ ^29^ ^]^ Accordingly, we have developed a DNA sequential logic circuit comprising an SR‐latch based on the NOR gate, an SR‐latch based on the NAND gate, and a D‐latch by utilizing a dynamic DNA/nicking enzyme (Nickase) Network Assembly toolbox.^[^ ^30^ ^]^ These circuits are characterized by their straightforward design, high autonomy, and reusability. Moreover, leveraging the remarkable biocompatibility of DNA circuits, we have successfully integrated these circuit devices into cells and the environment.

## 2 Results

### 2.1 Nicking‐Enzyme‐Driven Dynamic Displacement System

**Figure 1:**
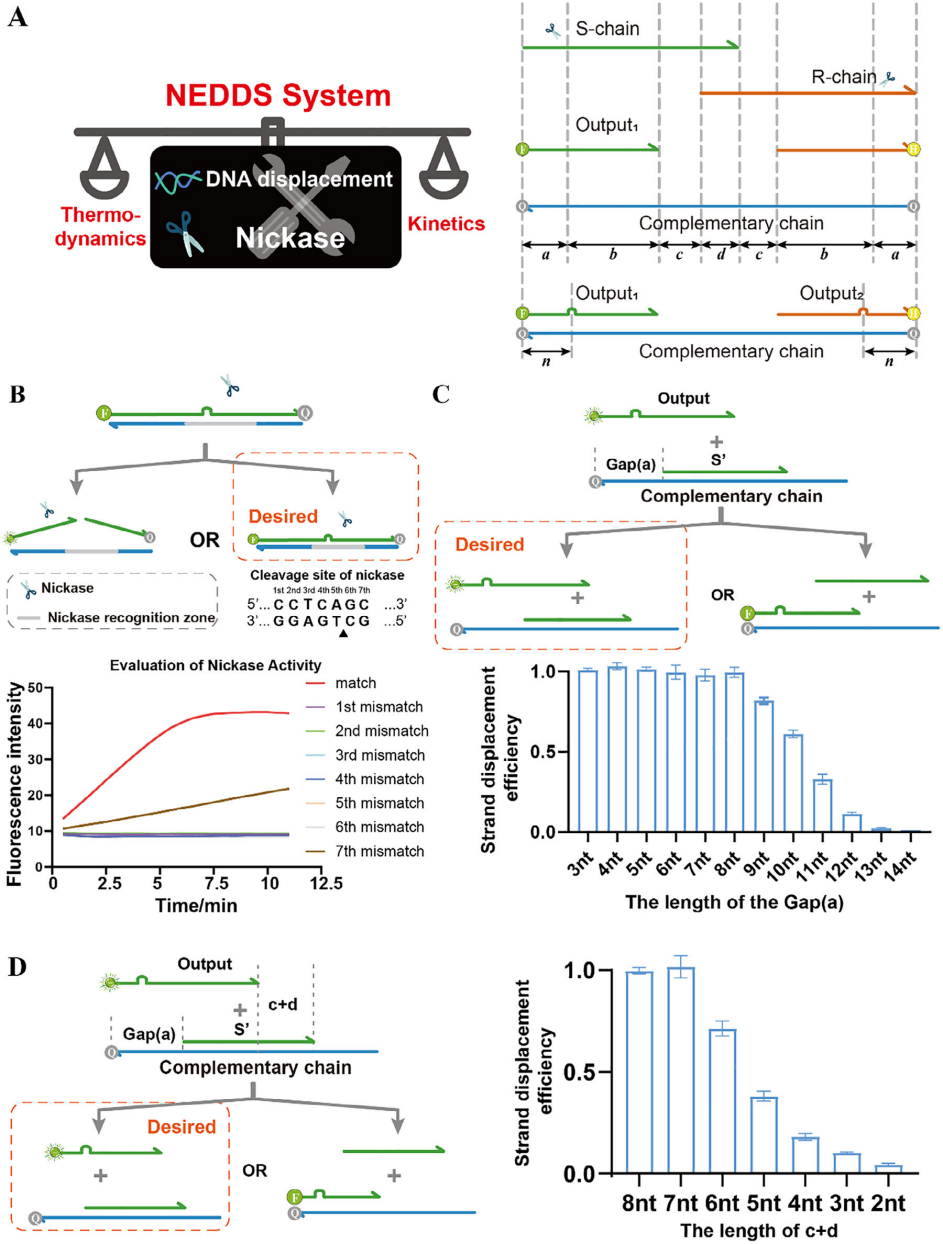
Nicking‐Enzyme‐Driven Dynamic Displacement System. A) Schematic diagram of the NEDDS. B) The effect of inhibition of cleavage activity of mispairing of different bases on the nickase recognition site. The results show that all the site mismatches except the seventh site mismatch on the nickase recognition site can completely inhibit the cleavage enzyme activity. C) Optimize the length of the gap exposed at the 3′ end of the complementary chain after the Input‐S has been cleaved by the nickase. The goal is that the Output strand cannot displace the cut Input‐S’. Strand displacement efficiency versus length, i.e., (experimental fluorescence intensity – negative control) / (positive control – negative control), Each experiment was repeated three times to calculate the fluorescence mean and standard deviation. D) Optimize the value of c+d. Each experiment was repeated three times to calculate the fluorescence mean and standard deviation.

We have constructed a Nicking‐Enzyme‐Driven Dynamic Displacement System (NEDDS, **Figure** **1**A) that realizes our needs by balancing thermodynamics and kinetics. Since the latch is an element with a bistable state, NEDDS consists of two parts that are symmetrical. The system contains five DNA strands: the S and R strands as input strands, the Output_1_ (O_1_), the Output_2_ (O_2_), and the complementary strand (C). They are divided into four parts, a, b, c, and d, according to the length of their complementary regions. We utilized the mismatch of the nickase recognition region to achieve the regulation of the nickase activity (Figure 1B) and set the distance of the mismatch site from the end of the strand as n.

We then optimized the length of the gap exposed at the 3′ end of the complementary chain after the Input‐S had been cleaved by the nickase (Figure 1C). The goal is that the Output strand cannot displace the cut Input‐S’. As shown by the results in Figure 1B, the mismatch between the Output and the complementary can be at any position on the nickase recognition region, except the seventh position. Thus, if the mismatch site on the Output_1_ is set n bases from the 5′ end. Due to the symmetry of the elements, the mismatch site on the Output_2_ is also n bases from the 3′ end. Since the mismatch site cannot be the seventh position of the nickase recognition region, this gives n‐5 ≤ a ≤ n at the 3′ end of Complementary, while n‐1 ≤ a ≤ n+4 at the 5′ end of Complementary. Therefore, the final result should be n‐1 ≤ a ≤ n. As shown in Figure 1C. Because the subsequent reaction requires the Input‐S’ to be displaced by the combined action of the Output_1_ and Input‐R, we need to realize the obstruction to the combination of the Output_1_ and the complementary with the Input‐S’ as short as possible. Therefore, we choose the gap length to be 8 nt, i.e., a = 8. To summarize, n can only be equal to 8 or 9.

Next, we optimized the value of c+d (Figure 1D). To ensure that the Input‐S’ is not displaced by the Output_1_, we optimized the length of the notch at the 5′ end of the Input‐S’ in Figure 1C, but it is also closely related to the 3′ length of the Input‐S’ (i.e., the c + d value). To subsequently ensure that the Input‐S’ can be displaced by both the Output and the Input‐R, the c+d value should be as small as possible, so we optimized c+d = 7. The optimization of c and d, respectively, is demonstrated in Figure S3 (Supporting Information).

The core function of this system is that the cut S' chain can prevent the Output_1_ from binding to the complementary chain, yet can be displaced by the Output chain together with the R chain. The results of the optimization show that although Length_a_ > Length_b+c_, ΔG_C‐O_ < ΔG_C‐S’_, and thermodynamics predicts that the O chain should replace the S’ chain to form a more stable double‐stranded structure, the reaction still fails to occur. We hypothesize that the thermodynamic drive is insufficient and that the high activation energy puts it into a kinetic bottleneck, preventing the reaction from occurring. Therefore, analyzed from a kinetic point of view, the reaction path of C‐S' + O → C‐O + S' can be divided into three steps: 1) Toehold region binding, 2) branch migration (BM), and 3) complete dissociation of the S' chain. The reaction rate is dominated by the slowest step (branch migration), which leads to the rate constant *k*_total_ ≈ *k*_BM_ ∝ *e* ^−(^ *^n^* ^⋅Δ^ *^G^* ^step+Δ^ *^G^* ^coop)/RT^ (Single‐step migration energy barrier, ΔG*_step_*; Cooperative Unbinding Energy, ΔG*_coop_*, n = b + c + d). The reaction rate tends to zero due to the longer b+c and higher ΔG*_coop_*, plus the lack of thermodynamic driving force, which makes the reaction “stagnant” in appearance. When the R chain is introduced, the ΔG*_coop_* of the reaction is lowered and the thermodynamic driving force is increased so that S' can be replaced. In conclusion, NEDDS regulates the thermodynamic and kinetic properties of the chain displacement reaction through the use of nickase to achieve the desired bistable function.

### 2.2 The SR‐Latch using NOR Gate

**Figure 2:**
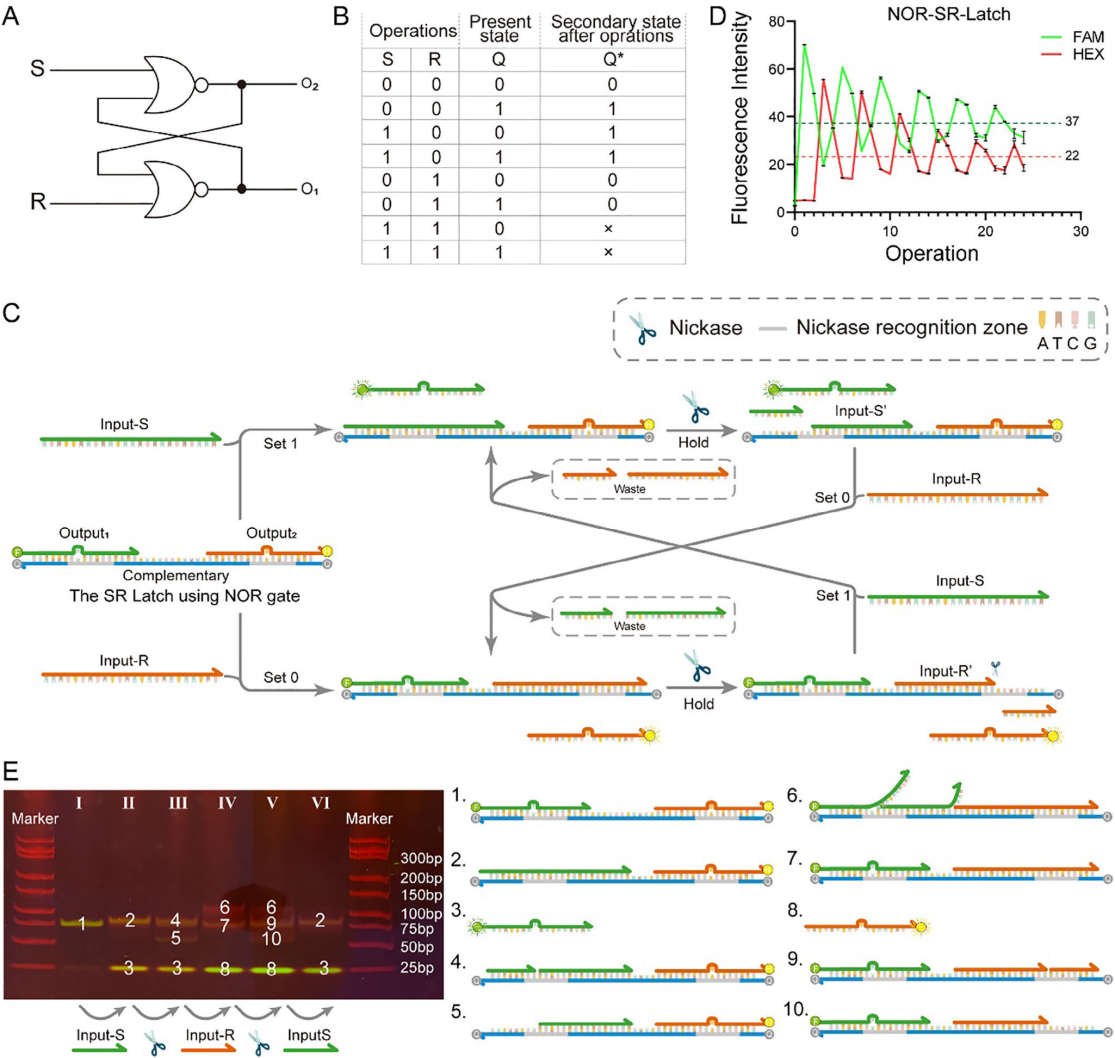
SR‐latch consisting of NOR‐gates. A) Circuit diagram. B) Truth table. C) Schematic diagram of the operation of SR‐latch consisting of NOR gates, including set 0, set 1, and hold functions. D) Functional verification result diagram of SR‐latch consisting of NOR gates. Each experiment was repeated three times to calculate the fluorescence mean and standard deviation. E) Validation of electrophoretic results of the reaction process of SR‐latch composed of NOR gates. The major component in each band is labeled with a number corresponding to the structure on the right side.

The SR latch consisting of NOR gates is first constructed using NEDDS. **Figure** **2**A,B depicts the circuit diagram and truth table of the SR‐latch constructed using NOR gates. To facilitate a concise description of the latch's state changes in response to inputs, we referred to the state before the input action as the present state (denoted by Q) and the state after the input action as the secondary state (denoted by Q*). In this context, we defined Output_1_ (O_1_) = 1 and Output_2_ (O_2_) = 0 to signify a stored data value of 1 (Q or Q* = 1). Conversely, when O_1_ = 0 and O_2_ = 1, it indicates a stored data value of 0 (Q or Q* = 0).

We designed and implemented a symmetric DNA device to realize the SR‐latch, consisting of three strands: Output_1_, Output_2_, and Complementary (see Figure 2C). In this setup, we assigned the 6‐Carboxyfluorescein (FAM) fluorophore to O_1_ and the Hexachlorofluorescein (HEX) fluorophore to O_2_. When the input is 1, we introduced the corresponding input strand, whereas when the input is 0, we removed the previously added input using a nickase (Nb.BbvCI). The binding of Output_1_ or Output_2_ to the Complementary strand prevents cleavage by the nickase due to a mismatch at the recognition site.

In the initial state, FAM is quenched by BHQ‐1, and HEX is quenched by BHQ‐2. When S = 1 and R = 0, the Input‐S is added without Input‐R, the Output_1_ is displaced, restoring the FAM's fluorescent signal. At this time, the secondary state of the latch, Q*, is set to 1 (O_1_ = 1, O_2_ = 0), indicating the activation of the “set 1” function (see Figure 2B,C). Conversely, when S = 0 and R = 1, the Input‐R is added without Input‐S, the Output_2_ is displaced, restoring the HEX's fluorescent signal. So, Q* is set to 0 (O_1_ = 0, O_2_ = 1), representing the activation of the “set 0” function (see Figure 2B,C). In the case of S = 0 and R = 0, the nickase is added without the input, and the existing Input‐S or Input‐R in the reaction system is removed by the nickase, the cut Input‐S’ or Input‐R’ cannot be replaced by Output_1_ or Output_2_ at this time, resulting in the latch assuming a “hold” function where Q* equals Q (see Figure 2B,C). The cut Intput‐S’ or Input‐R’ can only be replaced if “Output_1_ and Input‐R” or “Output_2_ and Input‐S” are present at the same time, while the FAM or HEX is quenched by the BHQ, making Output_1_ or Output_2_ = 0. When both S = 1 and R = 1, O_1_ and O_2_ simultaneously become 1, leading to a state that does not conform to the defined values of 0 or 1. This state is considered an error state. Consequently, in typical applications of the SR‐latch constructed with NOR gates, it is imperative to avoid having S = 1 and R = 1 simultaneously.

To assess the maximum performance capabilities under complex operations, we repeatedly executed the sequence: “set 1, hold, set 0, hold” to determine the latch's reuse limit. We established the following criteria for the experiment involving the SR‐latch utilizing a NOR gate. We formulated the rules for thresholding as follows: as the cycle proceeds, the first time a channel has a fluorescence value of 1 in theory that is less than 0 threshold or a fluorescence value of 0 in theory that is greater than 1 threshold stops the test. For each channel itself, the average of the minimum fluorescence value when the previous output is 1 and the maximum fluorescence value when the output is 0 is used as the threshold value. A detailed discussion of threshold value calculation can be found in Discussion S1 (Supporting Information). Figure 2D revealed that the device could maintain stability for 23 operations (approximately equal to 6 cycles). However, the main reason why the signal becomes fainter stems from the accumulation of waste. When the Latch performs the “Set 0/1” function, there is a competitive binding step where “Output1/2 + Input‐R/S” are expected to combine with the complementary chain. Since “Output1/2 + Input‐R/S” is longer than the waste, they win the initial competition. However, as the concentration of waste increases, the waste will begin to win and eventually completely dominate. As a result of this leakage, the memory will “plateau”. The application of magnetic beads may increase the lifetime of the latch by substantially reducing the degree of mixing in the system:^[^ ^31^ ^]^ the beads are trimmed from each chain of the initial element and retained by the magnetic field after each operation, while the waste is flushed away (Figure S4, Supporting Information). Furthermore, the reaction process and potential sources of leakage and crosstalk were investigated and confirmed using gel electrophoresis (Figure 2E). In the PAGE results, FAM and HEX exhibited distinct colors discernible to the naked eye. The 1st line of the PAGE is the latch itself, followed in turn by the addition of the Input‐S, nickase, Input‐R, nickase, and Input‐S to the previous channel to realize the operations “set 1, hold, set 0, hold, and set 1”.

### 2.3 The SR‐Latch using the NAND Gate

**Figure 3:**
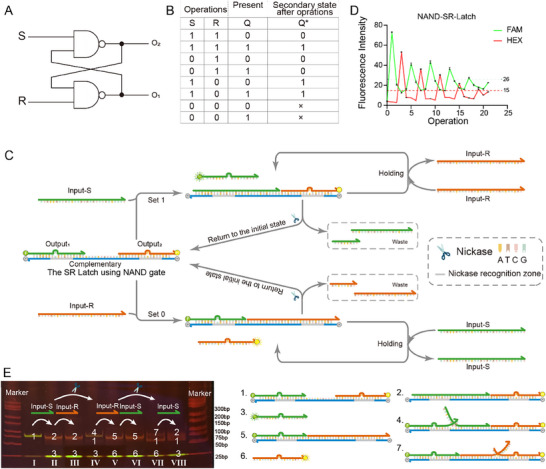
SR‐latch consisting of NAND‐gates. A) Circuit diagram. B) Truth table. C) Schematic diagram of the operation of SR‐latch consisting of NAND gates, including set 0, set 1, and hold functions. D) Functional verification result diagram of SR‐latch consisting of NAND gates. Each experiment was repeated three times to calculate the fluorescence mean and standard deviation. E) Validation of electrophoretic results of the reaction process of SR‐latch composed of NAND gates. The major component in each band is labeled with a number corresponding to the structure on the right side.

The SR latch consisting of NAND gates is next constructed using NEDDS without d‐region, and the circuit diagram and truth table are shown in **Figure** **3**A,B. We have modified the aforementioned symmetric DNA structure to achieve the desired function (see Figure 3C). The definition of 1 and 0 with different inputs and set 1 and 0 functions are similar to the SR‐latch using the NOR gate. When S = 1 and R = 1, the latch behaves as a bistable circuit where Q* is equal to Q (hold function, see Figure 3B,C). When S = 0 and R = 0, both O_1_ and O_2_ become 1 simultaneously, resulting in an error state. Therefore, in typical applications of the SR‐latch constructed with NAND gates, it is crucial to avoid the absence of both S and R simultaneously.

In the initial state, FAM is quenched by BHQ‐1, and HEX is quenched by BHQ‐2. When S = 1 and R = 0, the Input‐S is added without Input‐R, and the Output_1_ is displaced, restoring the FAM's fluorescent signal. At this time, the secondary state of the latch, Q*, is set to 1 (O_1_ = 1, O_2_ = 0), indicating the activation of the “set 1” function (see Figure 3B,C). Conversely, when S = 0 and R = 1, the Input‐R is added without Input‐S, the Output_2_ is displaced, restoring the HEX's fluorescent signal. So, Q* is set to 0, representing the activation of the “set 0” function (see Figure 3B,C). Before performing the “set” function, it is necessary to restore the component to its initial state, where the Input present in the system is removed by the nickase. The cut Input’ will become waste by dissociating from the complementary. For the hold function, take for example, the case of the hold function after “set 1”, when “Input_1_ and Output_2_” are bound to the Complementary, there is no toehold. So Input_2_ cannot displace Input_1_ and Output_2_ from the Complementary, and the hold function is realized. The same applies to the hold function after the “set 0”. Follow a threshold division criterion similar to that of the SR‐latch consisting of NOR gates. To assess the maximum performance capabilities under complex operations, we conducted repeated cycles of the following sequence: ″set 1, return to the initial state using a nickase, set 0, return to the initial state using a nickase″. Figure 3D demonstrated that the device could maintain stable operation for at least 20 operations (5 cycles). The hold function was also validated and corroborated in Figure S5 (Supporting Information). Additionally, the reaction process and potential sources of leakage and crosstalk were analyzed and confirmed through PAGE (Figure 3E). The 1st line of the PAGE is the latch itself. The 2nd and 4th lanes show the addition of Input‐S or nickase to the 1st lane system, respectively. The 3rd lane shows the addition of Input‐R within the 1st lane system. The 5th and 6th lanes show the addition of Input‐S or nickase to the 4th lane system, respectively. The 7th lane shows the addition of Input‐R within the 4th lane system. The 8th lane shows the addition of Input‐R within the 7th lane system. So Figure 3E shows the “set 1, return to the initial state using a nickase, set 0, return to the initial state using a nickase” cycle.

### 2.4 The D‐Latch

**Figure 4:**
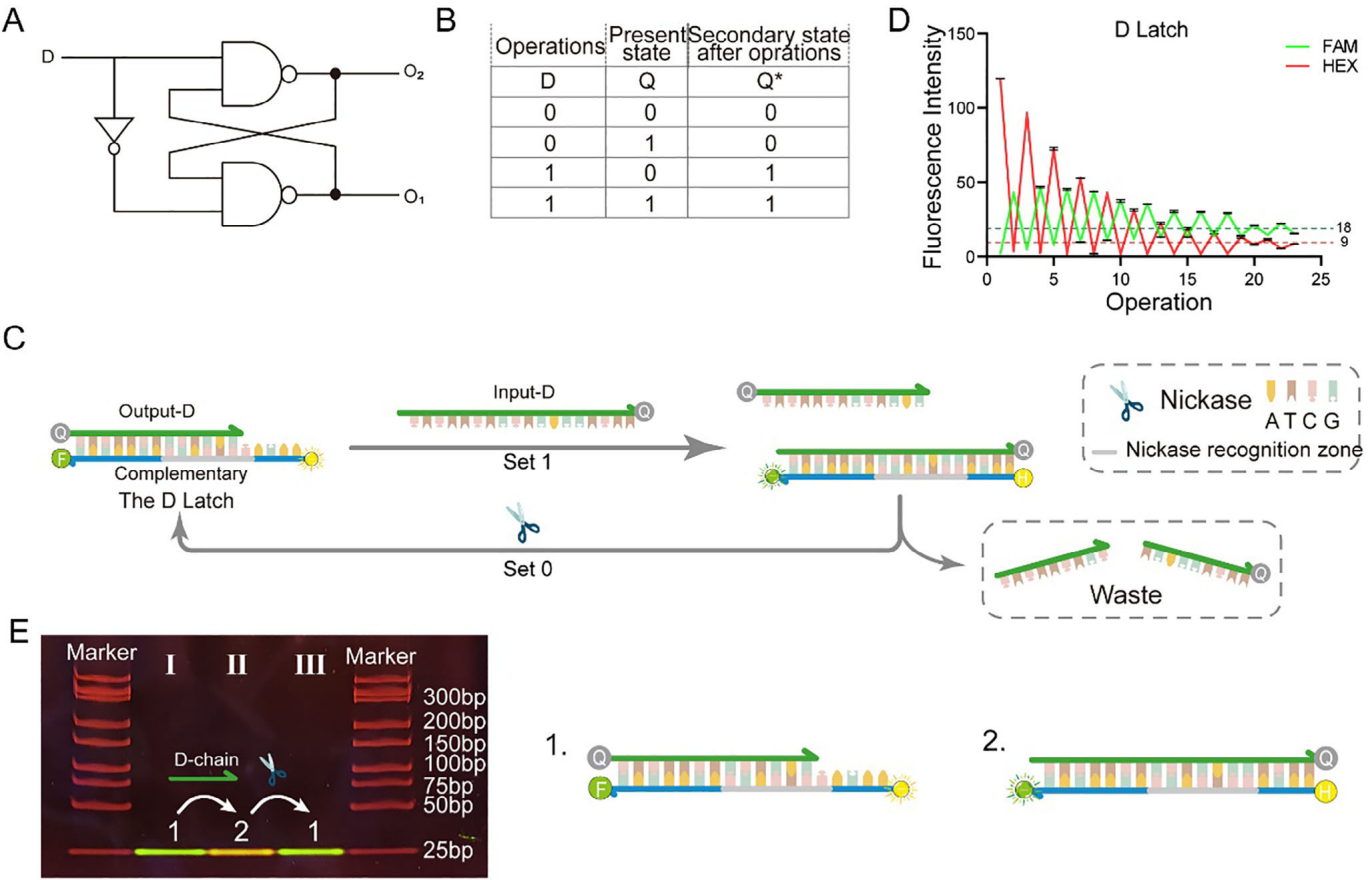
D‐latch. A) Circuit diagram. B) Truth table. C) Schematic diagram of D‐latch operation, including set 0 and set 1 functions. D) Functional verification result diagram of D‐latch. Each experiment was repeated three times to calculate the fluorescence mean and standard deviation. E) Validation of electrophoretic results of the reaction process of D‐latch.

As previously mentioned, simultaneous values of 1 for both S and R in the NOR gate‐based SR‐latch will result in an error. Similarly, an error occurs when both S and R are 0 in the NAND gate‐based SR‐latch. The D‐latch is introduced to prevent this error state, which addresses scenarios where the input can be both 0 and 1 simultaneously by incorporating a NOT gate at the input (see **Figure** **4**A). The definition of state and stored data value is similar to that of SR‐latch. The D‐latch is implemented by utilizing half of the NEDDS. In the initial state, FAM is quenched by BHQ‐1, and HEX is not quenched in the Output‐D/Complementary duplex. When D = 1, the Input‐D is added, and the Output‐D strand is displaced, restoring the FAM's fluorescent signal. The BHQ‐1 at the 3′ end of the Input‐D quenches HEX in the Complementary strand. So Q* is set to 1, indicating the activation of the “set 1” function. Conversely, when D = 0, the nickase is added to cleave the Input‐D. The Output‐D goes back to hybridize with Complementary, so the FAM is quenched and the HEX is not. So Q* is set to 0, representing the activation of the “set 0” function (see Figure 4B,C).

We conducted repeated cycles of the following sequence: “set 1, set 0” to explore the device's reuse limit. Figure 4D demonstrated that the device can maintain stable operation for at least 22 operations (11 cycles), following a threshold division criterion similar to that of the SR‐latch. Gel electrophoresis was further analyzed, and the reaction process was confirmed (Figure 4E). The 1st lane of the PAGE is the D‐latch itself. The 2nd lane shows the addition of Input‐D within the 1st lane system. The 3rd lane shows the addition of nickase within the 2nd lane system.

### 2.5 Addition Counter, Subtraction Counter, and Reversible Counter

**Figure 5:**
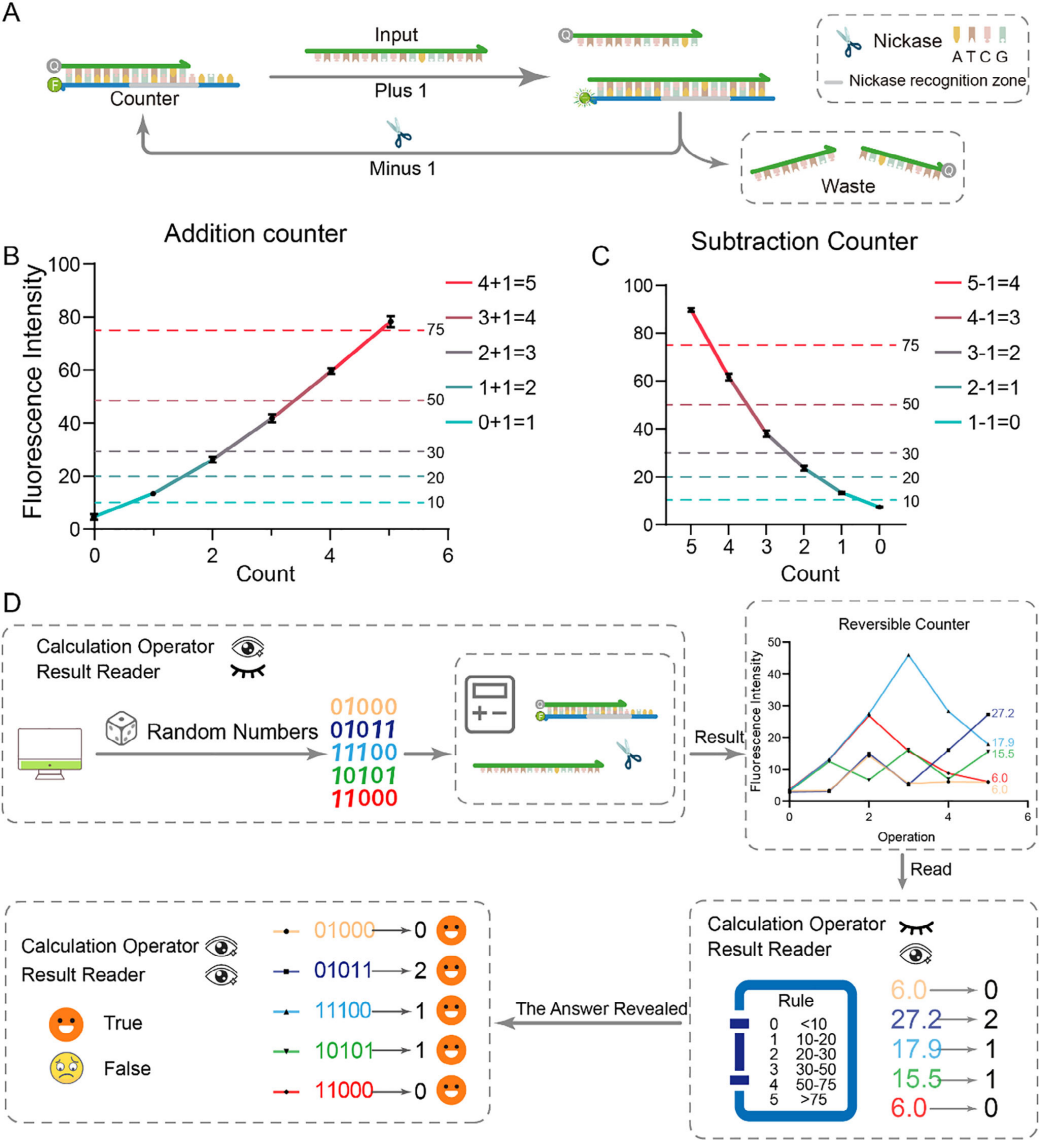
Counter based on the D‐latch. A) Schematic diagram of the reaction process of adding 1 and subtracting 1 in the counter. B) Functional verification and threshold division of addition counters. Each experiment was repeated three times to calculate the fluorescence mean and standard deviation. C) Functional validation and threshold division of subtraction counters. Each experiment was repeated three times to calculate the fluorescence mean and standard deviation. D) Blind verification of the function of the reversible counter. The equation is generated from random numbers, with 1 representing plus 1 and 0 representing minus 1. The calculation is performed by a calculation operator based on the equation. At the end of the calculation, the result is provided for the result reader (who has no prior knowledge of the equation) to read out the result, which is then compared with the result of the equation calculation.

Upon constructing the latch, we used D‐Latch to develop a counter (**Figure** **5**A). In this context, the device possesses complete memorability, achieved through a uniform input scheme. Addition 1 function involves introducing an input with a concentration of 1× (100 nM) into the system while subtraction 1 function entails adding 100 U mL^−1^ of Nb.BbvCI to the system and incubated for 7.5 min (The concentration and time optimization of the nicking enzyme is shown in Figure S6, Supporting Information), followed by inactivation at 85 °C for 25 min. The D‐Latch enables the completion of at least 22 counts in principle. To validate its functionality, we conducted experiments involving 5 counts. Initially, we determined the count thresholds by performing experiments with “0+1+1+1+1+1+1” and “5‐1‐1‐1‐1‐1‐1” (Figure 5B,C). Based on the results, we established the following thresholds: a fluorescence intensity below 10 corresponds to a count of 0; intensities between 10 and 20 indicate a count of 1; intensities between 20 and 30 represent a count of 2; intensities between 30 and 50 signify a count of 3; intensities between 50 and 75 denote a count of 4; and intensities above 70 correspond to a count of 5. Subsequently, we randomly selected five counting processes using a random number method, where a random number of 1 indicates an addition of 1, and a random number of 0 indicates a subtraction of 1. Besides that, when 0 is at the beginning of the random number process, or when the calculation result reaches 0, 0 cannot continue to perform the operation of minus 1. The functionality was verified through a blinded approach, wherein the counters and readers were different individuals, and the counting process remained undisclosed to the readers (Figure 5D). The results demonstrate that the reader could accurately interpret the results without knowing the specific counting process. This verifies the stability of our counter when performing successive mixed operations of addition and subtraction.

### 2.6 Biosensor and Biological Information Storage

**Figure 6:**
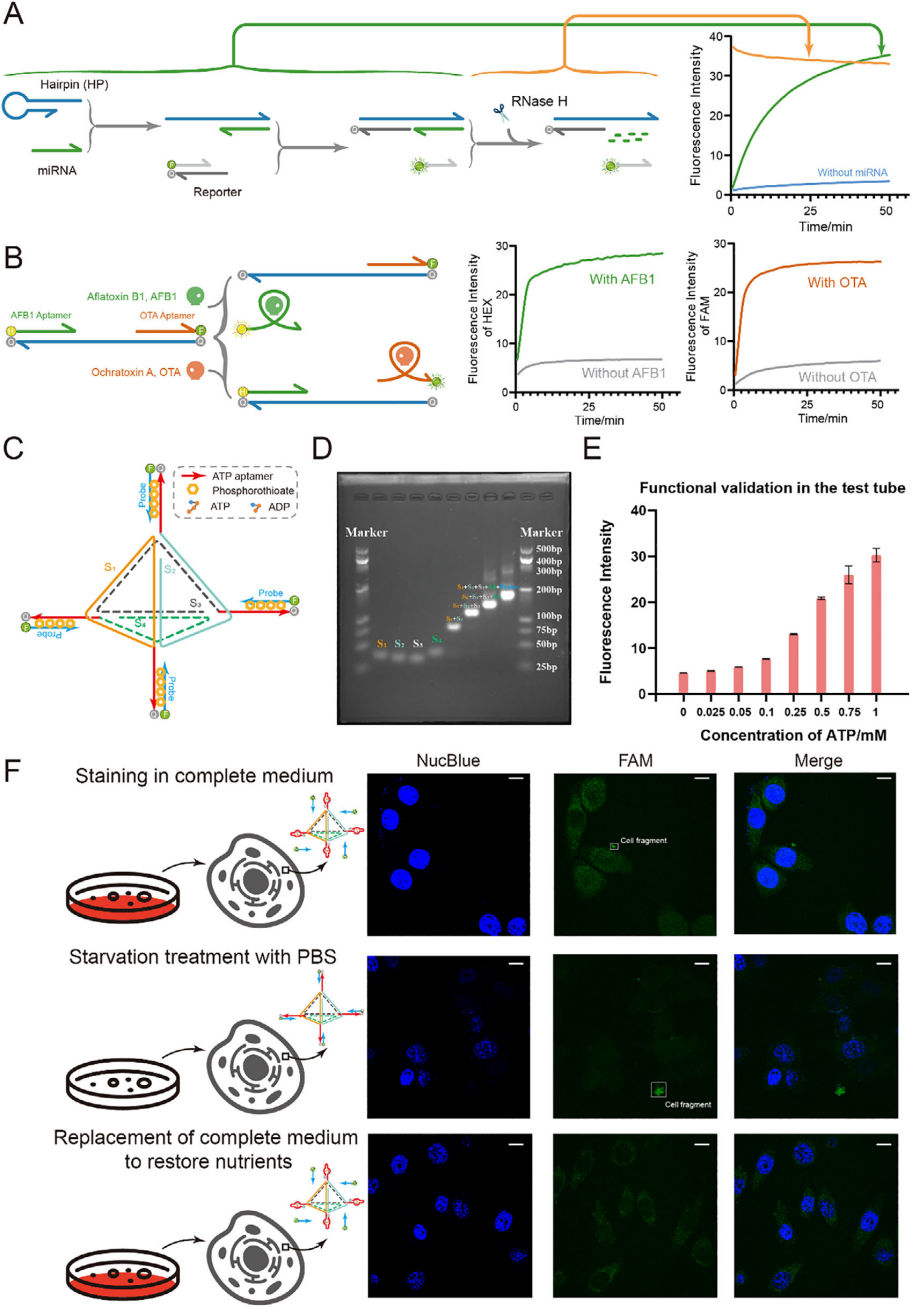
Applying latches to life environments to store bioinformation. A) SR‐latch consisting of NOR gates for miRNA monitoring. After the information is recorded, the record remains even though it disappears. Its functional test fluorescence profile is exhibited. B) SR‐latch consisting of NAND gates for monitoring toxins in the environment. The presence of any of the toxins is recorded and does not interfere with each other. C) Schematic diagram of combining the latch with a nucleic acid tetrahedron (the carrier into the cell, consisting of four strands, S_1_, S_2_, S_3,_ and S_4_). The 3′ end of S_1_, S_2_, S_3,_ and S_4_ is a section of ATP aptamer. The Probe strand is modified by thiolation to prevent its degradation by enzymes inside the cell. D) Electrophoretic verification of the structure of latches bound to nucleic acid tetrahedra. E) Functional validation of the “latch‐nucleic acid tetrahedron” complex in *vitro*. F) Functional validation of the “latch‐nucleic acid tetrahedron” complex in cells. From top to bottom are staining in complete medium, after 3 h of starvation treatment, and after replacing the complete medium. Scale bars are 10 µm.

To expand the scope of applications, we aimed to employ the storage function of latches within biological systems. To mimic the function of the SR‐Latch composed of NOR gates, we constructed a nucleic acid information recorder (**Figure** **6**A). We substituted Input‐S and Input‐R by the target nucleic acid strand (e.g., miRNA). Once the miRNA is present, it undergoes a strand displacement reaction with the hairpin and reporter to generate fluorescence (set1), which is permanently recorded and retained even if the miRNA is degraded by RNase H (hold). This corresponds exactly to the function of the SR‐latch consisting of a NOR gate, whose signal is preserved as long as the input has existed, even if the input disappears. When the miRNA strand was first degraded, it did not produce a signal (Figure S7, Supporting Information), suggesting that the fluorescence retention after RNase H degradation in Figure 6A is due to the storage function of the latch.

To mimic the function of the SR‐Latch consisting of NAND gates, we constructed an environmental toxin sensor to record the presence of the toxin and indicate environmental contamination (Figure 6B). We substituted Input‐S and Input‐R with the toxin (e.g., Aflatoxin B1 and Ochratoxin A). Once the toxins are present, they bind to the corresponding aptamers^[^ ^32^, ^33^ ^]^ and generate a fluorescent signal (set1). Even the presence of another toxin (Ochratoxin A) does not affect the output produced by the previous input (Aflatoxin B1) (hold). This design takes advantage of the function of the SR‐latch so that the presence of any of these toxins is indicative of environmental contamination.

Finally, we constructed the D‐Latch using ATP aptamers and introduced the D‐Latch into the cell by attaching it to a nucleic acid tetrahedron (Figure 6C). We replaced the Output‐D in the D‐Latch with Probe, and the thiolation keeps the Probe stable in the cell. ATP is also used to act as Input‐D. The detailed process by which DNA tetrahedra responds to ATP is shown in Figure S8 (Supporting Information). The inherent versatility of this structure enables a response to various biomolecules using aptamers (e.g., enzymes, proteins, and ions) or specific DNA/RNA fragments through strand replacement. We verified the structure through agarose gel electrophoresis (Figure 6D) and confirmed its functionality in *vitro* (Figure 6E). Subsequently, we introduced the structure into the cell, where it effectively fulfilled its role in storing and releasing information (Figure 6F). Co‐culturing the structure with cells while ensuring an adequate nutrient supply allowed the structure to enter the cells and bind to intracellular ATP releasing Probe to induce a fluorescent signal. The phosphorothioate modification of the Probe enables it to be stabilized without degradation while free in the cell. The D‐Latch set 1 function is realized at this time. Next, cells were starved by incubating them with PBS instead of the complete medium for 3 h. Due to nutrient deficiency, intracellular ATP concentration decreased. Then, ATP was converted to ADP and dissociated from the aptamer. Thus, the probe rebonded to the aptamer, and the fluorescence was quenched. Therefore, a significant decrease in intracellular fluorescence was observed, corresponding to the set 0 function of the D‐Latch. However, upon replenishing the cells with a complete medium, the cells regained nutrients. Then, ADP was converted to ATP and recombined with the aptamer, releasing the probe. The fluorescence increased again (set 1 function), indicating the structure's dynamic response to changes in ATP concentration within the cell (Figure 6F). The dynamic response to ATP concentration changes demonstrates the reproducibility, autonomy, and practical utility of the D‐latch.

Additionally, we noticed that cell fragments lacking their normal membrane structure exhibited enhanced and sustained staining. This experiment successfully demonstrates the utilization of DNA circuit components in a living environment. It paves the way for the realization of more intricate sequential logic circuits and showcases the biocompatibility advantages of DNA circuits, thereby highlighting their biological significance.

### 2.7 Discussion and Conclusion

DNA circuits hold immense potential for constructing computers using biological materials. However, most existing DNA circuits are categorized as combinational, with limited reports on sequential logic circuits. This scarcity seriously impedes the application and advancement of DNA circuits. Furthermore, numerous challenges persist even in the domain of sequential logic circuits. For instance, the components lack inherent memory and rely on human intervention for sequential functionality. Additionally, the complex design of these components renders them non‐reusable; while they can store information, they cannot release it, among other deficiencies. We compare several components that have the potential to implement DNA temporal logic circuits in Table S2 (Supporting Information).^[^ ^24^, ^25^, ^26^, ^27^, ^28^, ^34^ ^]^

The sequential logic circuit we implemented is a DNA circuit that can transition between two states, much like a dissipative system. However, dissipative systems usually transition continuously between two states,^[^ ^35^, ^36^, ^37^, ^38^ ^]^ whereas sequential logic circuits have a hold function in addition to that. If the sequential logic circuit is not in a convertible state, it will not be able to switch states even if a downstream input is present. Reusability is the basis for realizing the conversion function. We realized the reusability function through the sequential reaction of nickase and DNA strand replacement, in which the cleavage site of nickase and the length of the toehold of strand replacement were carefully designed to realize the adjustability of the hold‐and‐set function. The transition function is implemented in such a way that a circuit part representing one state (e.g., the left half of the SR‐latch using NOR gate, Figure 2C) is returned to the initial state, while a circuit part representing another state (e.g., the right half of the SR‐latch using NOR gate, Figure 2C) is activated. This is different from that reusability where the circuit returns to the initial state completely after the circuit is computed.^[^ ^39^, ^40^, ^41^ ^]^

For the biosensor's potential for environmental toxin monitoring, In our experiments, the fluorescence signal stabilized within 5–10 min after toxin introduction, a time range comparable to or faster than many existing aptamer sensors (e.g., electrochemical sensor: 30–180 min^[^ ^42^, ^43^, ^44^, ^45^ ^]^; Fluorescence: 5–60 min^[^ ^46^, ^47^, ^48^ ^]^). Since the latch is not connected to a signal amplification system, its limit of detection (Figure S9, Supporting Information) is not better than the existing sensors, but its set 1, set 0, and, hold functions could not be realized by the existing sensors. Improving the latch's sensitivity is one of the directions of our subsequent efforts.

There has also been some advanced research into good implementations of temporal logic circuits. Although both types of circuits are involved in dealing with time‐related content, they differ in their focus and application areas: sequential logic circuits are more concerned with state transitions and storage in circuits, while temporal logic circuits are more focused on dealing with the temporal relationships between events.^[^ ^34^, ^49^ ^]^ Sequential logic circuits have an inherent memory capability to store historical states and determine outputs based on current inputs and the previous state. For example, an SR latch can maintain its output state even after the input signal is removed. This memory capability is critical for applications that require state retention, such as counters or data storage systems. In contrast, temporal logic circuits focus on time‐dependent relationships between events rather than state storage. They analyze sequences of inputs that vary over time to enforce logical constraints (e.g., “Event A must occur before Event B”). Although both circuit types involve temporal elements, sequential logic emphasizes state transitions and memory, while temporal logic prioritizes event ordering and temporal constraints.

We devised a straightforward and versatile structure to facilitate the construction of a latch. This approach capitalizes on the inherent capabilities of biological components such as DNA and enzymes. By ensuring that memory is integrated within the components, we achieved uniformity across human operations, enabling component reusability for a minimum of 20 cycles. Furthermore, we extended the application of latches by constructing addition, subtraction, and reversible counters, thereby confirming their robustness. Lastly, we successfully introduced these circuit components into a living environment, validating their functionality and manifesting DNA circuits' biological potential.

## 3 Experimental Section

### Materials and Apparatus

All oligonucleotides used in this study were synthesized and purified by Tsingke Biotechnology Co., Ltd. (Wuhan, China) and Sangon Biotech Co., Ltd. (Shanghai, China). Nt.BbvCI nicking enzyme (Nt.BbvCI), 10× rCutsmart buffer (1× rCutsmart buffer, 50 mM Potassium Acetate, 20 mM Tris‐acetate, 10 mM Magnesium Acetate, 100 µg ml^−1^ Recombinant Albumin, pH 7.9) were purchased from New England Biolabs Inc. (Beijing, China). Agarose, 5× TBE Buffer, 4S Red Plus Nucleic Acid Stain, 6× DNA Loading Dye, and DNAMarker A (25–500 bp), Native PAGE Preparation kit, RNase H (100U), 10× RNase H Buffer (500 mM Tris‐HCl, 750 mM KCl, 30 mM MgCl2, 100 mM DTT, pH 8.3) were purchased from Sangon Biotech Co., Ltd. (Shanghai, China). Ochratoxin A and Aflatoxin B1 were purchased from Aladdin Biochemical Technology Co., Ltd. (Shanghai, China). Phosphate buffered solution (PBS, PH 7.2–7.4, 0.01 M) was bought from the Solarbio Science & Technology Co., Ltd. (Beijing, China). The concentration of DNA oligonucleotides was measured using a NanoDrop 2000 UV–vis spectrophotometer (Thermo Fisher Scientific, Waltham, MA, USA). The sequences of all oligos are listed in Table S1 (Supporting Information). The secondary structure of Latches at 37 °C was predicted by NUPACK (https://nupack.org/).^[^ ^50^ ^]^

### Cell Lines and Cell Culturing

Human SW480 cells were purchased from the American Type Culture Collection (ATCC, Virginia, America). SW480 cells were cultured in DMEM basal medium (Thermo Fisher, Shanghai, China), at 37 °C in a 5% (v/v) CO2 incubator. FBS (10%, v/v; Gibco, Thermo Fisher Scientific) was added to the DMEM.

### Fluorescence Monitoring

Fluorescence was measured using a Rotor‐Gene Q2 plex HRM Instrument (QIAGEN, Hilden, Germany) at 37 °C. The fluorescence intensity was recorded every 30 s. The excitation and emission wavelengths were set to 470 and 510 nm for the FAM channel and 530 and 555 nm for the HEX channel.

### Agarose Gel Electrophoresis Analysis

Agarose gel electrophoresis was carried out using a 3% agarose gel at 110 V in a 1× TBE buffer. Then, 10 µL of sample solution and 2 µL of loading buffer were mixed and added to each well. After the separation in the electrophoresis apparatus (Junyi, Beijing, China), the gels containing DNA were stained using 4S Red Plus and visualized at a wavelength of 590 nm using a Bio‐Rad Universal Hood II gel imaging system (Bio‐Rad, Shanghai, China).

### Polyacrylamide Gel Electrophoresis (PAGE) Characterization of the Reaction Process

15% polyacrylamide gel was used to characterize the occurrence of the experiment. Electrophoresis was carried out at 150 V for 30 min. After separation, the gel was stained with 4S Red Plus for 60 min on a shaking table and imaged by a fluorescence gel imaging system (Blue Light Gel Imager, G500312, Sangon Biotech Co. Ltd). Pictures were imaged by a mobile phone camera.

### Preparation for the Reaction

Latches, addition counter, subtraction counter, reversible counter, and DNA tetrahedron were annealed by heating to 95 °C for 10 min followed by cooling to 37 °C over 60 min in 1× rCutsmart Buffer, ensuring that they can form a stable and desirable secondary structure. Reagents for the reaction and the detection of the products were prepared in one vial. Enzymes were added to the vial last, and the fluorescence intensity was recorded immediately after enzyme addition.

### Optimization of the Length of Each Region of the Latch

The 20 µL reaction system consisted of 100 nM Complementary, 100 nM Output, 100 nM S', and 1× rCutsmart Buffer. The Complementary strand and S' were previously warmed and annealed into a stable secondary structure. Output was additionally added to measure fluorescence. The length of the S' was different for each group.

### Evaluation of Nickase Recognition Site Mismatches

The 20 µL reaction system consisted of 100 nM Complementary, 100 nM match or mismatch strands, 500 U mL^−1^ Nt.BbvCI and 1× rCutsmart Buffer. Nt.BbvCI was not the only nickase available in the NEDDS system. By simple modification of the sequence of the cleavage site in NEDDS, it can be replaced with any other nickase that is active at 37 °C.

### The SR‐Latch using NOR Gate

The initial 50 µL reaction system consisted of 200 nM NOR‐SR‐Latch (composed of SR‐NOR‐Output_1_, SR‐NOR‐Output_2_, and SR‐NOR‐Complementary). Then 1 µL of 10 µM SR‐NOR‐S or SR‐NOR‐R, 1 µL of 10 000 U mL^−1^ Nt.BbvCI was added as needed. The system was replenished with 0.5 µL water and 0.5 µL 10× rCutsmart buffer after every four operations. The Nt.BbvCI was inactivated at 85 °C for 25 min when needed.

### The SR‐Latch using the NAND Gate

The initial 50 µL reaction system consisted of 200 nM NAND‐SR‐Latch (composed of SR‐NAND‐Output1, SR‐NAND‐Output2, and SR‐NAND‐Complementary). Then 1 µL of 10 µM SR‐NAND‐S or SR‐NAND‐R, 1 µL of 10 000 U mL^−1^ Nt.BbvCI was added as needed. The system was replenished with 0.5 µL water and 0.5 µL 10× rCutsmart buffer after every four operations. The Nt.BbvCI was inactivated at 85 °C for 25 min when needed.

### The D‐Latch

The initial 50 µL reaction system consisted of 200 nM D‐Latch (composed of D‐Output1 and D‐Complementary). Then 1 µL of 10 µM Input‐D, 1 µL of 10 000 U mL^−1^ Nt.BbvCI was added as needed. The system was replenished with 0.5 µL water and 0.5 µL 10× rCutsmart buffer after every four operations. The Nt.BbvCI was inactivated at 85 °C for 25 min when needed.

### Subtractive Counter Nickase Concentration Optimization

The 50 µL reaction system consisted of 500 nM subtraction counter (composed of Counter‐Complementary and Counter‐Output), 500 nM Counter‐Input, 1× rCutsmart Buffer, and 0, 20, 50, 100, or 200 U mL^−1^ Nt.BbvCI.

### Addition Counter, Subtraction Counter, and Reversible Counter

The initial 50 µL system consisted of 500 nM addition counter, subtraction counter or reversible counter, and 1× rCutsmart Buffer. Then, 1 µL of 5 µM Input was added to the system for plus 1 reaction. For minus 1 reaction, 0.5 µL of 25 000 U mL^−1^ Nb.BbvCI was added to the system and incubated for 7.5 min, followed by inactivation (85 °C, 25 min).

### Nucleic Acid Information Recorder

The 20 µL reaction system consisted of 200 nM Hairpin, 200 nM miRNA for 1× concentration, 200 nM Reporter or 0.01 U µL^−1^ RHase H and 1× RNase H Buffer.

### Toxin Sensors

The 20 µL reaction system consisted of 200 nM sensor (AFB1 Aptamer, OTA Aptamer, and complementary strands), 50 µg mL^−1^ AFB1 or 50 µg mL^−1^ OTA, and 1× Buffer (10 mM Tris‐HCl (pH 7.5), 50 mM MgCl2, 50 mM NaCl).

### Biological Information Storage

Aptamer‐DNA tetrahedron was self‐assembled by mixing the component strands in 1× rCutsmart Buffer. SW‐480 cells were cultured in 15 mm glass bottom cell culture dishes and incubated for 12 h with 5% CO_2_. Then, the prepared aptamer‐DNA tetrahedron was added to dishes with a final concentration of 150 nM, and the cells were incubated with the aptamer‐DNA tetrahedron at 37 °C for 3 h. Starvation treatment was taken by replacing the complete medium with PBS and incubating for 3 h. After starvation treatment, the PBS was replaced with the complete medium. Cells were fixed with 4% paraformaldehyde and then sealed using a sealer with Dapi dye. Then, the cells were imaged using a confocal laser‐scanning microscope (OLYMPUS FV1000, Tokyo, Japan).

## Conflict of Interest

The authors declare no conflict of interest.

## Author Contributions

T.X., C.L., and M.H. contributed equally to this work. T.X., T.W., C.L., and M.H. conceived the study. T.W. led the project. T.X. designed the experiment, analyzed the experiment data, and performed most of the experiments. X.Z., J.X., Z.Z., and Z.W. participated in the experiment and discussion. T.X., C.L., and M.H. contributed equally. T.X. drafted the paper. T.W. and Z.W. revised the paper.

## Supporting information



Supporting Information

## Data Availability

The data that support the findings of this study are available in the supplementary material of this article.

## References

[1] L. Qian , E. Winfree , Science 2011, 332, 1196. 21636773 10.1126/science.1200520

[2] H. Su , J. Xu , Q. Wang , F. Wang , X. Zhou , Nat. Commun. 2019, 10, 5390. 31772166 10.1038/s41467-019-13310-2 PMC6879481

[3] T. Song , A. Eshra , S. Shah , H. Bui , D. Fu , M. Yang , R. Mokhtar , J. Reif , Nat. Nanotechnol. 2019, 14, 1075. 31548688 10.1038/s41565-019-0544-5

[4] S. Garg , S. Shah , H. Bui , T. Song , R. Mokhtar , J. Reif , Small 2018, 14, 1801470. 10.1002/smll.201801470 30022600

[5] X. W. Xiong , M. S. Xiao , W. Lai , L. Li , C. H. Fan , H. Pei , Angew. Chem., Int. Ed. 2021, 60, 3397. 10.1002/anie.202013883 33350563

[6] M. Hu , X. Cheng , T. Wu , Nucleic Acids Res. 2024, 52, 7384. 38828769 10.1093/nar/gkae470 PMC11229313

[7] R. Fu , J. Hou , Z. Wang , Y. Xianyu , ACS Nano 2024, 18, 14754. 38781600 10.1021/acsnano.4c04265

[8] R. Lopez , R. Wang , G. Seelig , Nat. Chem. 2018, 10, 746. 29713032 10.1038/s41557-018-0056-1

[9] C. Zhang , Y. Zhao , X. Xu , R. Xu , H. Li , X. Teng , Y. Du , Y. Miao , H. C. Lin , D. Han , Nat. Nanotechnol. 2020, 15, 709. 32451504 10.1038/s41565-020-0699-0

[10] A. A. Tregubov , P. I. Nikitin , M. P. Nikitin , Chem. Rev. 2018, 118, 10294. 30234291 10.1021/acs.chemrev.8b00198

[11] X. D. Lin , Y. Q. Liu , J. K. Deng , Y. L. Lyu , P. C. Qian , Y. F. Lia , S. Wang , Chem. Sci. 2018, 9, 1774. 29675221 10.1039/c7sc05246d PMC5892130

[12] Q. Q. Hu , H. Li , L. H. Wang , H. Z. Gu , C. H. Fan , Chem. Rev. 2019, 119, 6459. 29465222 10.1021/acs.chemrev.7b00663

[13] S. X. Jiang , Z. L. Ge , S. Mou , H. Yan , C. H. Fan , Chem 2021, 7, 1156.

[14] L. Yang , Q. Tang , M. Zhang , Y. Tian , X. Chen , R. Xu , Q. Ma , P. Guo , C. Zhang , D. Han , Nat. Commun. 2024, 15, 4583. 38811607 10.1038/s41467-024-48869-y PMC11136972

[15] Y. Zhang , Y. Guo , H. Yang , X. Miao , Q. Feng , Biosens. Bioelectron. 261, 116520. 10.1016/j.bios.2024.116520 38924812

[16] H. Sun , D. Zhao , Y. He , H. M. Meng , Z. Li , Adv. Sci. 2024, 11, 2402531. 10.1002/advs.202402531 PMC11321679 38864341

[17] J. Cavanagh , Sequential Logic: Analysis and Synthesis, CRC Press, Boca Raton, Florida, USA, 2018.

[18] W. J. Dally , R. C. Harting , Digital Design: A Systems Approach, Cambridge University Press, Cambridge, UK, 2012.

[19] E. Lipiansky , Electrical, Electronics, and Digital Hardware Essentials for Scientists and Engineers, John Wiley & Sons, Hoboken, NJ, USA, 2012.

[20] D. Chen , Z. Wu , X. Xu , S. Yang , Chemistry 2020, 26, 13235. 32337743 10.1002/chem.202001240

[21] B. Daly , T. S. Moody , A. J. M. Huxley , C. Yao , B. Schazmann , A. Alves‐Areias , J. F. Malone , H. Q. N. Gunaratne , P. Nockemann , A. P. de Silva , Nat. Commun. 2019, 10, 49. 30664631 10.1038/s41467-018-07902-7 PMC6341106

[22] X. J. Jiang , D. K. P. Ng , Angew. Chem., Int. Ed. 2014, 53, 10481. 10.1002/anie.201406002 25078949

[23] G. Strack , M. Ornatska , M. Pita , E. Katz , J. Am. Chem. Soc. 2008, 130, 4234. 18321113 10.1021/ja7114713

[24] W. Tang , W. Zhong , J. Fan , Y. Tan , Q. Huang , Y. Liu , Chem. Commun. 2019, 55, 6381. 10.1039/c9cc02632k 31089654

[25] Z. Guo , X. Zhang , S. H. Zhou , New J. Chem. 2023, 47, 8925.

[26] D. Scalise , M. Rubanov , K. Miller , L. Potters , M. Noble , R. Schulman , ACS Synth. Biol. 2020, 9, 749. 32212717 10.1021/acssynbio.9b00398

[27] C. Zhang , L. Shen , C. Liang , Y. Dong , J. Yang , J. Xu , ACS Appl. Mater. Interfaces 2016, 8, 9370. 26990044 10.1021/acsami.6b00847

[28] J. Zhao , A. Pokhilko , O. Ebenhöh , S. J. Rosser , S. D. Colloms , Nucleic Acids Res. 2019, 47, 4896. 30957849 10.1093/nar/gkz245 PMC6511857

[29] T. Xie , Y. Deng , J. Zhang , Z. Zhang , Z. Hu , T. Wu , Nucleic Acids Res. 2022, 50, 8431. 35904810 10.1093/nar/gkac650 PMC9410916

[30] A. Baccouche , K. Montagne , A. Padirac , T. Fujii , Y. Rondelez , Methods 2014, 67, 234. 24495737 10.1016/j.ymeth.2014.01.015

[31] B. Wang , S. S. Wang , C. Chalk , A. D. Ellington , D. Soloveichik , Proc. Natl. Acad. Sci. U S A 2023, 120, 2217330120. 10.1073/pnas.2217330120 PMC10500265 37669382

[32] G. Xu , J. Zhao , H. Yu , C. Wang , Y. Huang , Q. Zhao , X. Zhou , C. Li , M. Liu , J. Am. Chem. Soc. 2022, 144, 7731. 35442665 10.1021/jacs.2c00478

[33] G. Xu , C. Wang , H. Yu , Y. Li , Q. Zhao , X. Zhou , C. Li , M. Liu , Nucleic Acids Res. 2023, 51, 7666. 37351632 10.1093/nar/gkad541 PMC10415127

[34] M. Zhang , C. Yancey , C. Zhang , J. Wang , Q. Ma , L. Yang , R. Schulman , D. Han , W. Tan , Sci. Adv. 2024, 10, adn3329. 10.1126/sciadv.adn3329 PMC10997190 38578999

[35] Z. Zhou , Y. Ouyang , J. Wang , I. Willner , J. Am. Chem. Soc. 2021, 143, 5071. 33755445 10.1021/jacs.1c00486

[36] Q. Liu , H. Li , B. H. Yu , Z. J. Meng , X. M. Zhang , J. B. Li , L. F. Zheng , Adv. Funct. Mater. 2022, 32, 2201196.

[37] E. Del Grosso , P. Irmisch , S. Gentile , L. J. Prins , R. Seidel , F. Ricci , Angew. Chem., Int. Ed. 2022, 61, 202201929. 10.1002/anie.202201929 PMC9324813 35315568

[38] E. Del Grosso , E. Franco , L. J. Prins , F. Ricci , Nat. Chem. 2022, 14, 600. 35668213 10.1038/s41557-022-00957-6

[39] X. Liu , Z. Chen , K. Wan , Y. Luo , J. Yang , L. Li , K. Tao , X. Xiao , M. Zhang , ACS Nano 2025, 19, 9906. 40035235 10.1021/acsnano.4c15176

[40] Y. V. Gerasimova , D. M. Kolpashchikov , Angew. Chem., Int. Ed. 2016, 55, 10244. 10.1002/anie.201603265 27430161

[41] A. Eshra , S. Shah , T. Q. Song , J. Reif , IEEE Trans. Nanotechnol. 2019, 18, 252.

[42] F. Jahangiri‐Dehaghani , H. R. Zare , Z. Shekari , Talanta 2024, 266, 124947. 37459787 10.1016/j.talanta.2023.124947

[43] H. Zhang , S. Ye , L. Huang , S. Fan , W. Mao , Y. Hu , Y. Yu , F. Fu , Anal. Methods 2022, 15, 99. 36484245 10.1039/d2ay01682f

[44] D. Ciobanu , O. Hosu‐Stancioiu , G. Melinte , F. Ognean , I. Simon , C. Cristea , Biosensors 2023, 14, 7. 38248384 10.3390/bios14010007 PMC10813172

[45] F. Jahangiri‐Dehaghani , H. R. Zare , Z. Shekari , Food Anal. Methods 2022, 15, 192.

[46] D. I. Meira , A. I. Barbosa , J. Borges , R. L. Reis , V. M. Correlo , F. Vaz , Crit. Rev. Food Sci. Nutr. 2024, 64, 6318. 36688280 10.1080/10408398.2023.2168248

[47] Y. Liu , D. Liu , C. Li , S. Cui , Z. Yun , J. Zhang , Y. Wei , F. Sun , Crit. Rev. Food Sci. Nutr. 2022, 64, 5515. 36519502 10.1080/10408398.2022.2155107

[48] A. Hitabatuma , Y. H. Pang , L. H. Yu , X. F. Shen , Food Chem. 2021, 342, 128303. 33158674 10.1016/j.foodchem.2020.128303

[49] A. P. Lapteva , N. Sarraf , L. Qian , J. Am. Chem. Soc. 2022, 144, 12443. 35785961 10.1021/jacs.2c04325 PMC9284558

[50] J. N. Zadeh , C. D. Steenberg , J. S. Bois , B. R. Wolfe , M. B. Pierce , A. R. Khan , R. M. Dirks , N. A. Pierce , J. Comput. Chem. 2011, 32, 170. 20645303 10.1002/jcc.21596

