# Supplementary material for: DNA Sequential Logic Circuits for Reversible Counters and Dynamic Biomolecular Sensing
Source: Adv Sci (Weinh). 2025 Jun 19;12(32):e05793. doi: 10.1002/advs.202505793 (PMC12407326; doi:10.1002/advs.202505793)
Supplement: Supplementary file 1 — Supporting Information [file ADVS-12-e05793-s001.docx]

**Supplementary Data**

**DNA Sequential Logic Circuits for Reversible Counters and Dynamic Biomolecular Sensing**

Tianci Xie^a,b,c,1^, Changjiang Li^b,1^, Minghao Hu^b,1^, Xingyu Zhong^d^, Junbin Xiao^b^, Zhen Zhang^e^, Ze Wang^a,^*, Tongbo Wu^b,^*

^a^ Orthopedics Department, Wuhan Children's Hospital (Wuhan Maternal and Child Healthcare Hospital), Tongji Medical College, Huazhong University of Science & Technology, Wuhan, 430015, China.

^b^ School of Pharmacy, Tongji Medical College, Huazhong University of Science and Technology, Wuhan, 430030, China.

^c^ GI Cancer Research Institute, Tongji Hospital, Tongji Medical College, Huazhong University of Science and Technology, Wuhan, 430030, China.

^d^ Department and Institute of Urology, Tongji Hospital, Tongji Medical College, Huazhong University of Science and Technology, Wuhan, 430030, China.

^e^ The First Affiliated Hospital, and College of Clinical Medicine, Henan University of Science and Technology, Luoyang, China, 471003

* To whom correspondence should be addressed. Email: [wutongbo@hust.edu.cn](mailto:wutongbo@hust.edu.cn) and [13296628594@163.com](mailto:13296628594@163.com)

^1^ These authors contributed equally: Tianci Xie, Changjiang Li, Minghao Hu.

**Supplementary figures**


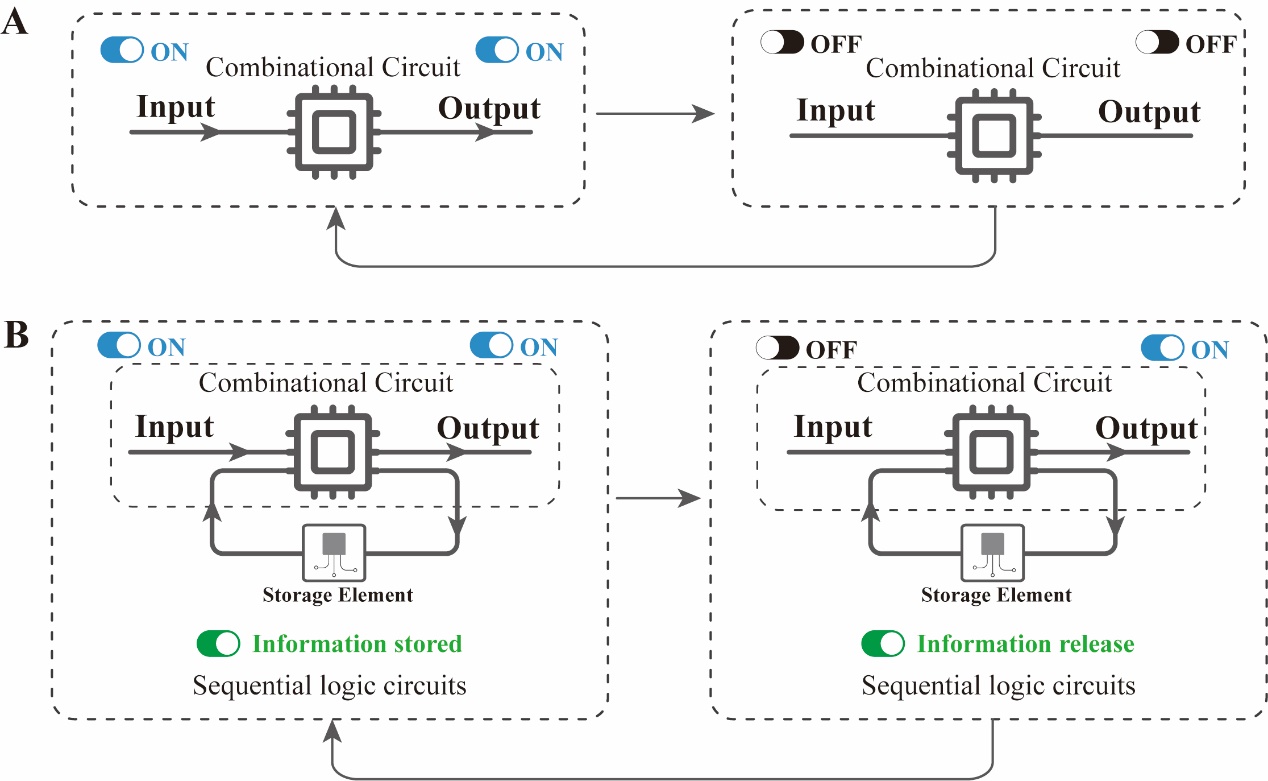


Supplementary Figure S1. Schematic diagram of Combinational Logic Circuit and Sequential Logic Circuit. (A) The operation of combinational logic circuits requires a continuous input and once aborted, the information is lost. (B) Sequential logic circuits consist of combinational logic circuits and storage elements. The output of the sequential logic circuit is determined by both the input and the information stored in the storage element. Even though there is no input, an output can be made based on the information stored in the storage element.


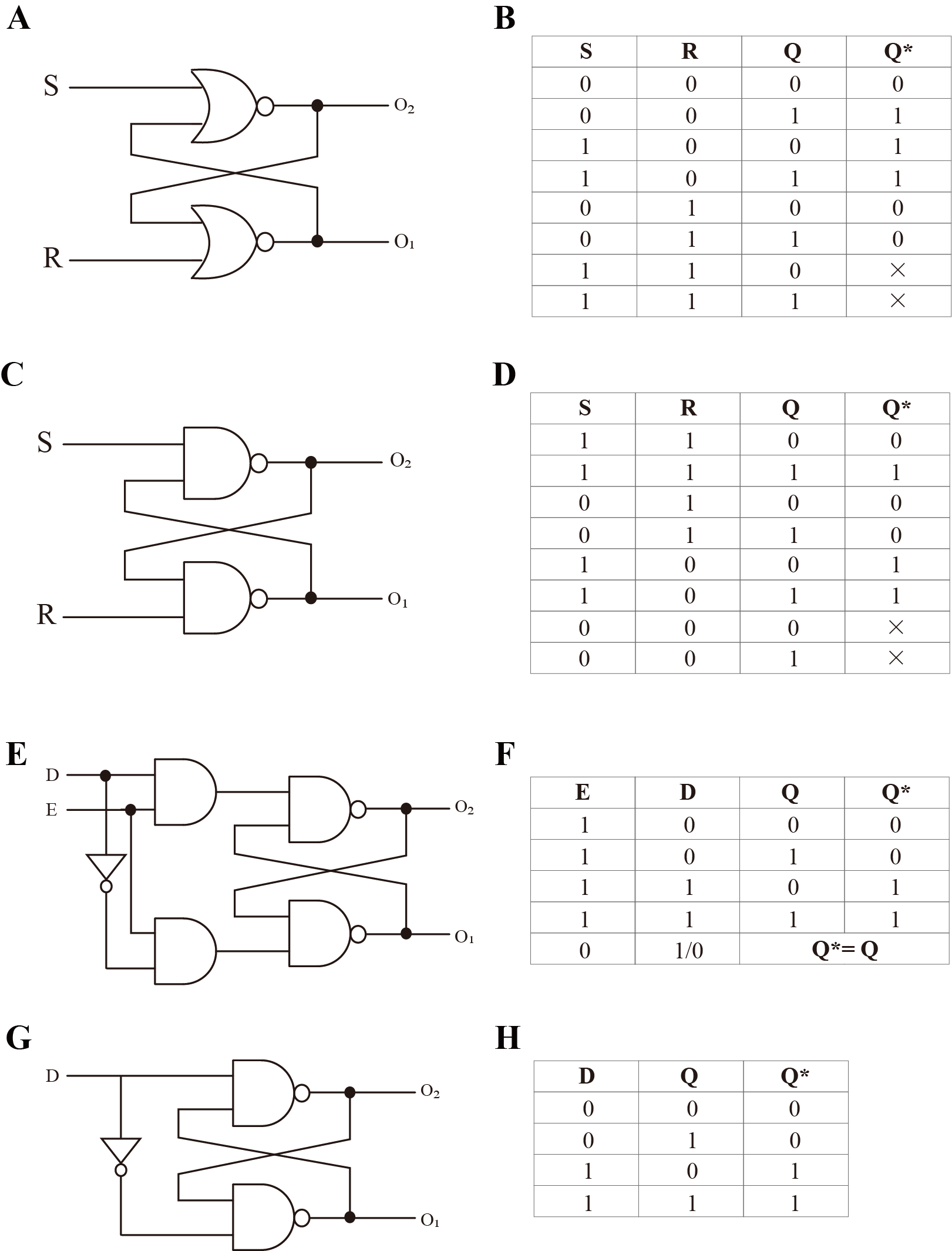


Supplementary Figure S2. Circuit diagram and truth table for the latch. In order to conveniently describe the relationship between the change of state of the latch under the input, the state of the latch before the action of the input is called the present state, denoted by Q, and the state after the action of the input is called the secondary state, denoted by Q*. Accordingly, defining Output_1_ (O_1_) =1 and Output_2_ (O_2_) =0 indicates that the stored data is 1 (Q or Q*=1), and defining O_1_=0 and O_2_=1 indicates that the stored data is 0 (Q or Q*=0). × represents the "holding or undefined" state. (A) Circuit diagram of the SR-latch consisting of the NOR-gate. (B) Truth table of the SR-latch consisting of the NOR-gate. (C) Circuit diagram of the SR-latch consisting of the NAND-gate. (D) Truth table of the SR-latch consisting of the NAND-gate. (E) Circuit diagram of the D-latch. (F) Truth table of the D-latch. (G) Circuit diagram of the simplified D-latch. (H) Truth table of the simplified D-latch.


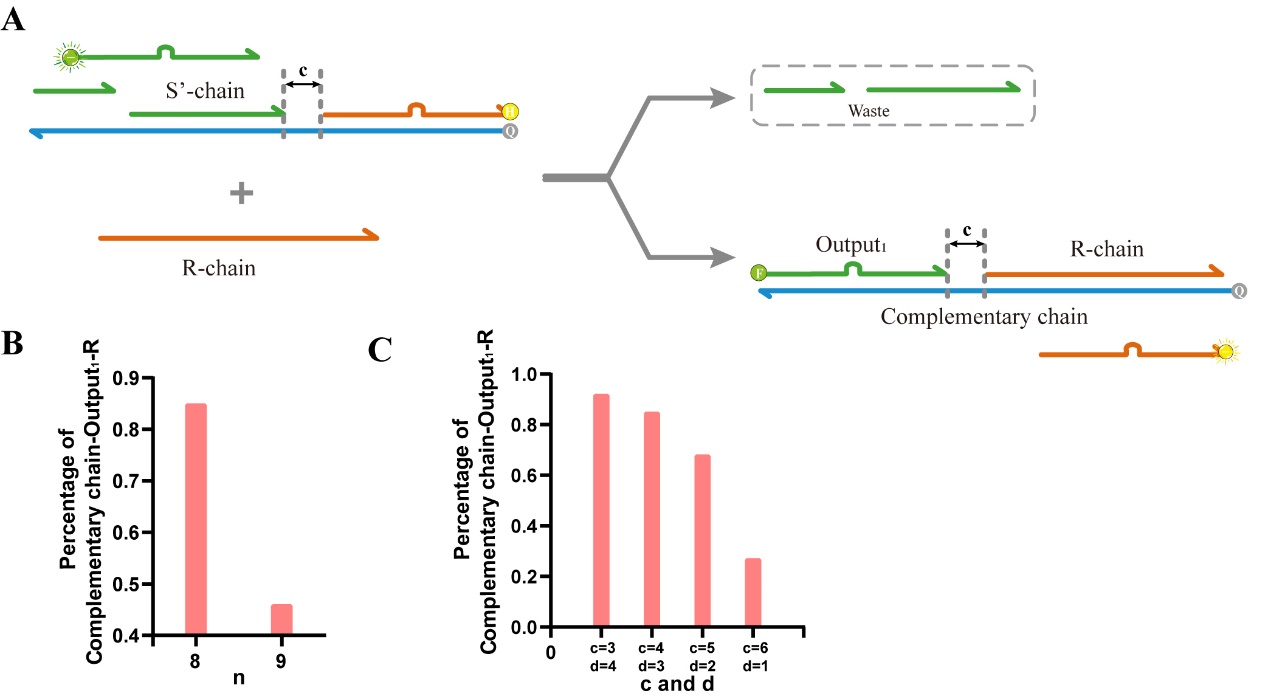


Supplementary Figure S3. Choose the value of n, c, and d. (A) Schematic diagram of the optimized reaction. (B, C) NUPACK was utilized to predict the reaction process in which the Output_1_, together with the Input-R, displaces the cleaved Input-S’. (B) In summary, we choose n = 8. (C) Considering that area c is the toehold area for subsequent strand replacement, it should not be too short, so c=4, d=3 is chosen.


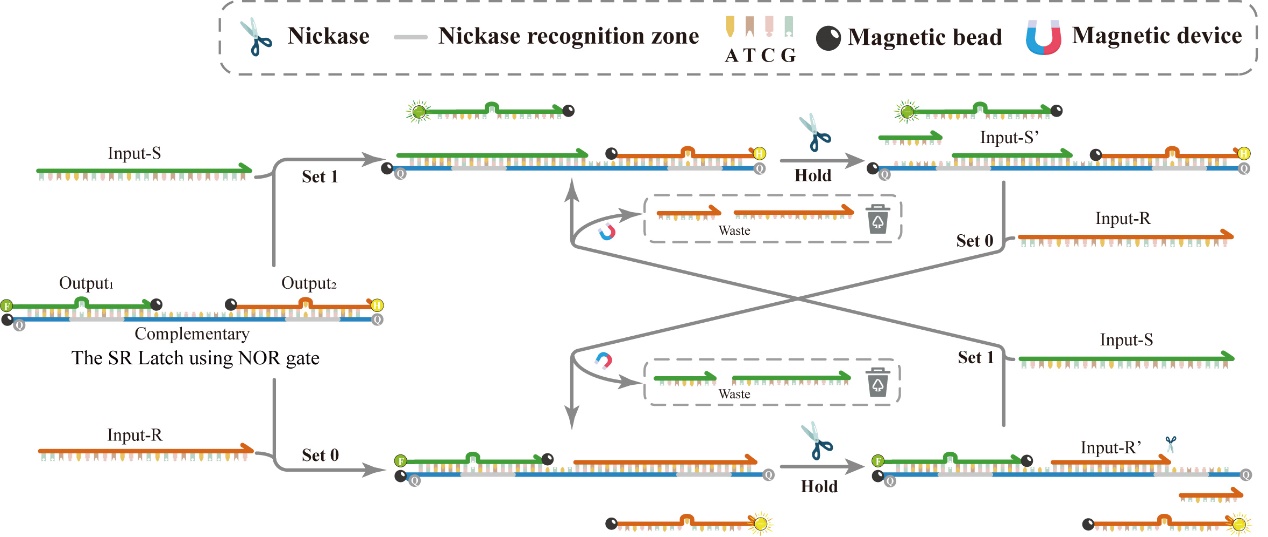


Supplementary Figure S4. Schematic diagram of the SR-latch consisting of NOR gates that utilize magnetic beads to remove waste.


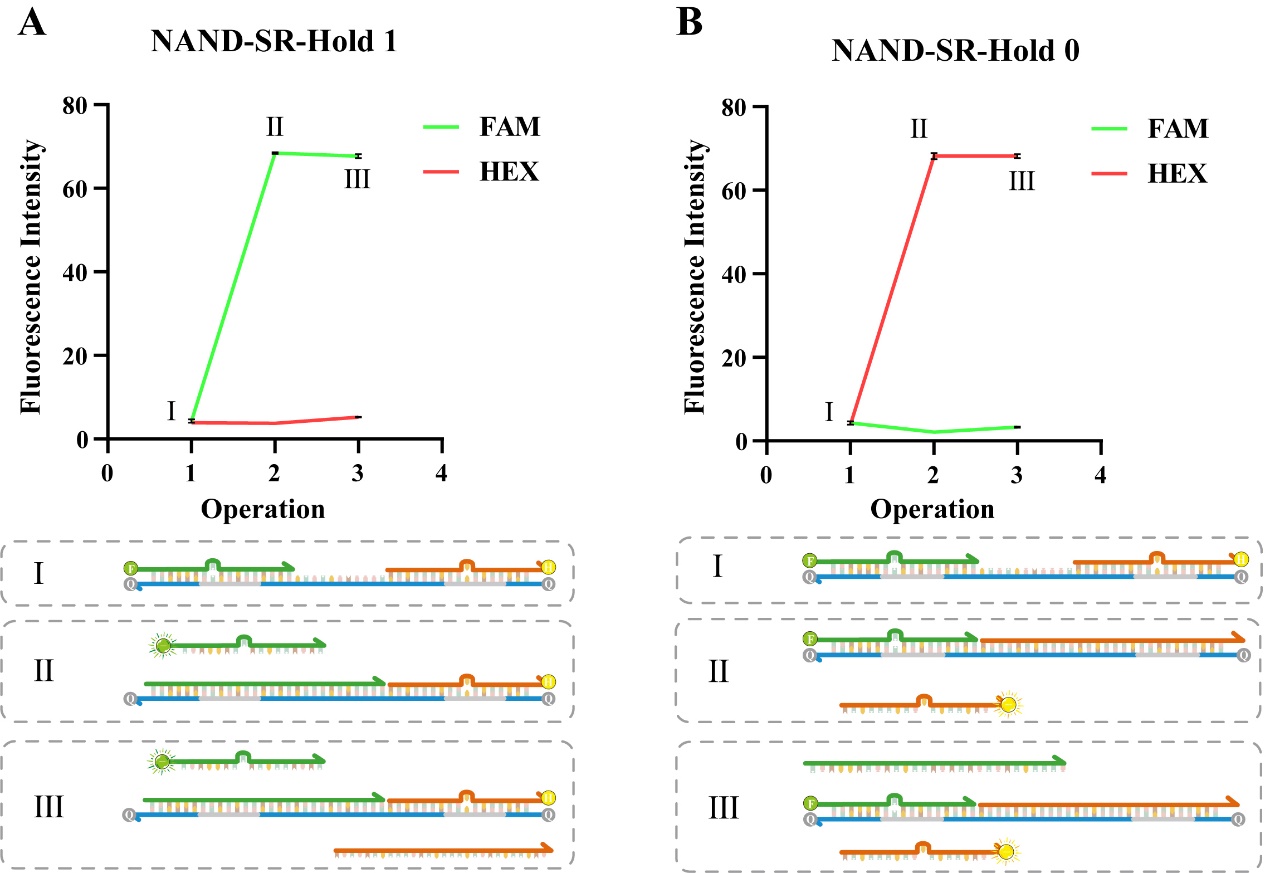


Supplementary Figure S5. NAND-SR-Latch hold function. (A) Hold 1. (B) Hold 0.


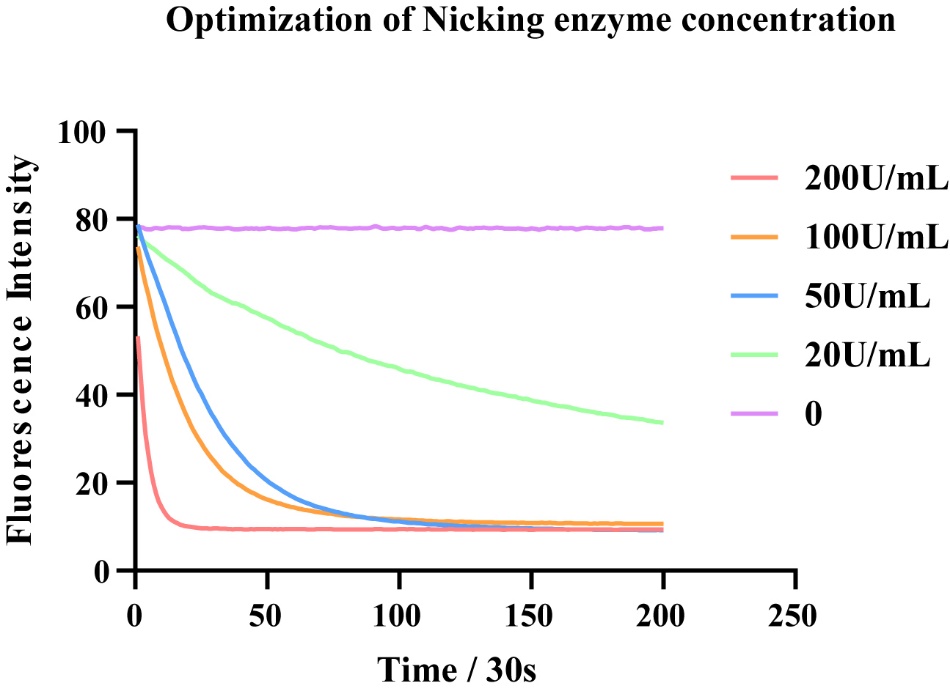


Supplementary Figure S6. Optimization of Nicking Enzyme concentration in subtractive methods. We chose its concentration to be 100 U/mL.


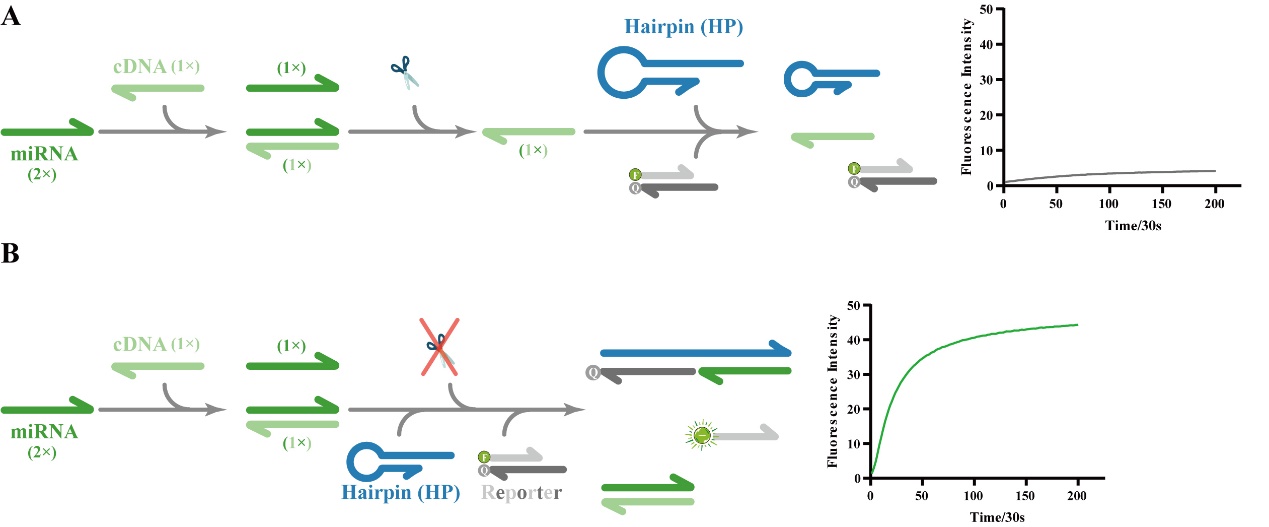


Supplementary Figure S7. (A) The RNA degradation process does not trigger the hairpin. (B) The presence of the free target RNA can trigger the hairpin.


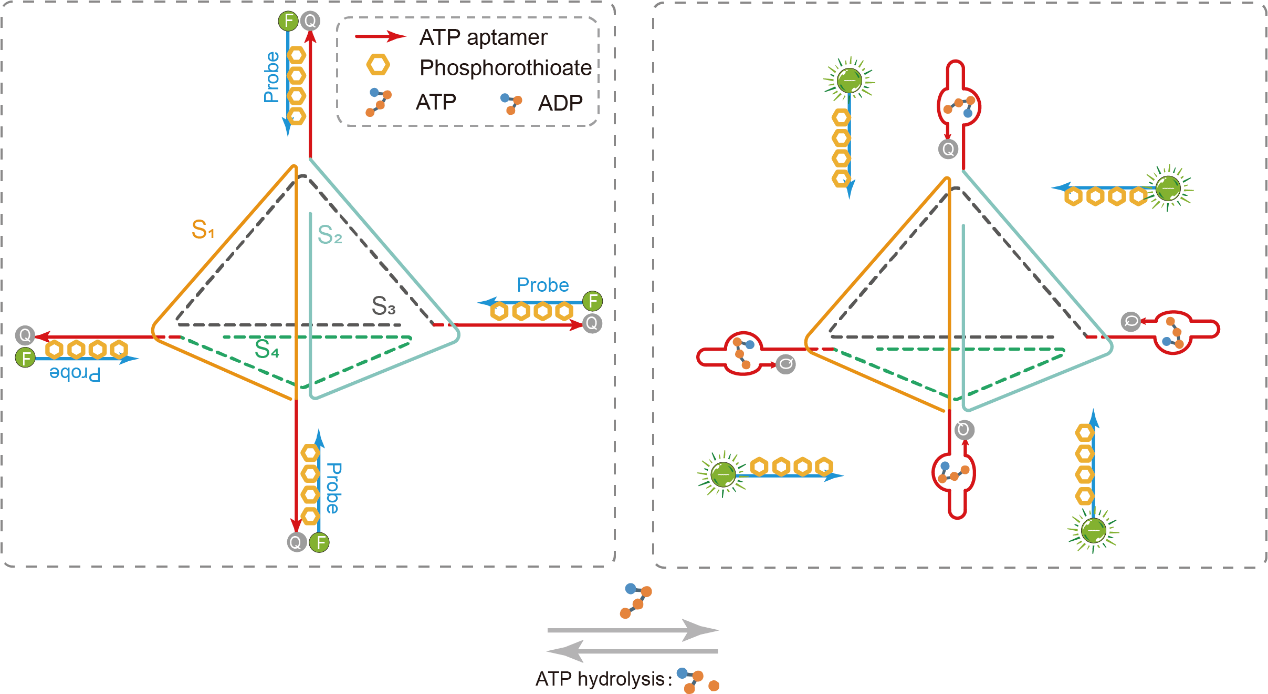


Supplementary Figure S8. Detailed process of DNA tetrahedron response to ATP.


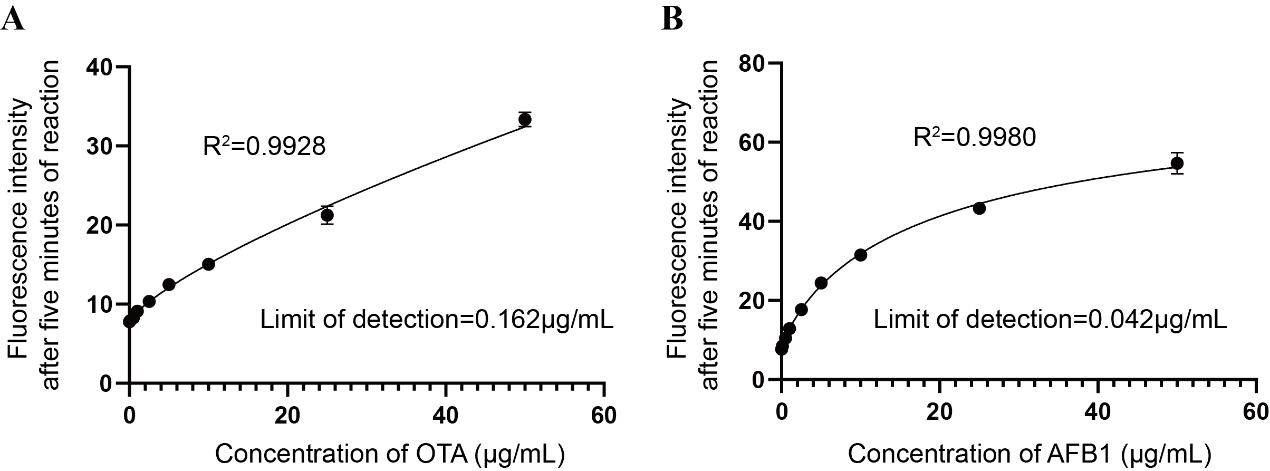


Supplementary Figure S9. Standard curves and detection limits for (A) OTA and (B) AFB1. Detection limits were obtained by the 3-fold signal-to-noise ratio method.

**Supplementary tables**

**Supplementary Table S1** Sequences of the oligonucleotides used in this work

| **Name** | **Sequences (5’-3’)** |
| --- | --- |
| **The SR-Latch using NOR gate** | |
| **SR-NOR-Output_1_** | FAM-GCTAATGGTGAGGTCTG |
| **SR-NOR-Output_2_** | CTACTGAGCAGAGGATC-HEX |
| **SR-NOR-Complementary** | BHQ1-GATCCTCAGCTCAGTAGAGTGACCGAGACAGACCTCAGC  ATTAGC-BHQ1 |
| **SR-NOR-S** | GCTAATGCTGAGGTCTGTCTCGGT |
| **SR-NOR-R** | GGTCACTCTACTGAGCTGAGGATC |
| **The SR-Latch using NAND gate** | |
| **SR-NAND-Output_1_** | FAM-GCTAATGGTGAGGTCTG |
| **SR-NAND-Output_2_** | TGAGGGAGCAGAGGATC-HEX |
| **SR-NAND-Complementary** | BHQ1-GATCCTCAGCTCCCTCAGCCCTCAGCGACAGACCTCAGC  ATTAGC-BHQ1 |
| **SR-NAND-S** | GCTAATGCTGAGGTCTGTCGCTGAGGGC |
| **SR-NAND-R** | TCGCTGAGGGCTGAGGGAGCTGAGGATC |
| **The D-Latch** | |
| **D-Complementary** | HEX-AAGACCTCAGCAGAAGAG-FAM |
| **D-Output** | BHQ1-CTCTTCTGCTGAG |
| **D-D chain** | CTCTTCTGCTGAGGTCTT-BHQ1 |
| **Addition counter, subtraction counter and reversible counter** | |
| **Counter-Complementary** | AAGACCTCAGCAGAAGAG-FAM |
| **Counter-Output** | BHQ1-CTCTTCTGCTGAG |
| **Counter-Input** | CTCTTCTGCTGAGGTCTT |
| **Nucleic acid information recorder** | |
| **Hairpin** | TCAACATCAGTCTGATAAGCTAGATACTGGTTCGCTATAGAGTGCTTATCAGACTGA |
| **miRNA-21** | UAGCUUAUCAGACUGAUGUUGA |
| **cDNA** | TCAACATCAGTCTGATAAGCTA |
| **Reporter-FAM** | FAM-GGTTCGCTATAGAGTGCTTAT |
| **Reporter-BHQ** | AGTCTGATAAGCACTCTATAGCGAACC-BHQ1 |
| **Toxin sensor** | |
| **Complementary** | TGTCCGATGCTTTTTTTTTTTTTTTTTTTTTTTTTTTTTTTACAACACGTGCAC |
| **AFB1-Aptamer** | GTGCACGTGTTGTCTCTCTGTGTCTCGTGC |
| **OTA-Aptamer** | GATCGGGTGTGGGTGGCGTAAAGGGAGCATCGGACA |
| **Bioinformation (ATP) storage** | |
| **S1** | ACATTCCGTCTGAAACATTACATGCTACACGAGAAGAGCCATAGTATTTTTTACCTGGGGGAGTATTGCGGAGGAAGGT-BHQ1 |
| **S2** | TATCACCCAGTTGACAGTGTAGCATGTAATAGATGCGAGCCAATACTTTTTTACCTGGGGGAGTATTGCGGAGGAAGGT-BHQ1 |
| **S3** | TCAACTGGGTGATAAAACGACACTTGGGAATCTACTATGGCTCTTCTTTTTTACCTGGGGGAGTATTGCGGAGGAAGGT-BHQ1 |
| **S4** | TTCAGACGGAATGTGCTTCCCAAGTGTCGTTTGTATTGGCTCGCATTTTTTTACCTGGGGGAGTATTGCGGAGGAAGGT-BHQ1 |
| **Probe** | FAM-A*C*C*T*T*C*C*T*C*C*G*C*A*A (*-Phosphorothioate) |
| **Supplementary Figure S3. Evaluation of nicking enzyme** | |
| **Nickase-Complementary** | FAM-AAGAGCTGAGGAGAA-BHQ |
| **Nickase-match** | TTCTCCTCAGCTCTT |
| **Nickase-1st mis** | TTCTGCTCAGCTCTT |
| **Nickase-2nd mis** | TTCTCGTCAGCTCTT |
| **Nickase-3rd mis** | TTCTCCACAGCTCTT |
| **Nickase-4th mis** | TTCTCCTGAGCTCTT |
| **Nickase-5th mis** | TTCTCCTCTGCTCTT |
| **Nickase-6th mis** | TTCTCCTCACCTCTT |
| **Nickase-7th mis** | TTCTCCTCAGGTCTT |
| **Supplementary Figure S5. Optimize the length of the gap** | |
| **Gap-Complementary** | CTGATAAGCTAGAAGACCCGAGACAGAAGTGAGCCTCAGC-BHQ1 |
| **Gap-Output** | FAM-GCTGACGCTCACTTCTG |
| **Gap-S’-3nt** | GAGGCTCACTTCTGTCTCGGGT |
| **Gap-S’-4nt** | AGGCTCACTTCTGTCTCGGGT |
| **Gap-S’-5nt** | GGCTCACTTCTGTCTCGGGT |
| **Gap-S’-6nt** | GCTCACTTCTGTCTCGGGT |
| **Gap-S’-7nt** | CTCACTTCTGTCTCGGGT |
| **Gap-S’-8nt** | TCACTTCTGTCTCGGGT |
| **Gap-S’-9nt** | CACTTCTGTCTCGGGT |
| **Gap-S’-10nt** | ACTTCTGTCTCGGGT |
| **Gap-S’-11nt** | CTTCTGTCTCGGGT |
| **Gap-S’-12nt** | TTCTGTCTCGGGT |
| **Gap-S’-13nt** | TCTGTCTCGGGT |
| **Gap-S’-14nt** | CTGTCTCGGGT |
| **Supplementary Figure S6. Optimize the value of c+d** | |
| **c+d-Complementary** | CTGATAAGCTAGAAGACCCGAGACAGAAGTGAGCCTCAGC-BHQ1 |
| **c+d-Output** | FAM-GCTGACGCTCACTTCTG |
| **c+d-8nt** | TCACTTCTGTCTCGGGT |
| **c+d-7nt** | TCACTTCTGTCTCGGG |
| **c+d-6nt** | TCACTTCTGTCTCGG |
| **c+d-5nt** | TCACTTCTGTCTCG |
| **c+d-4nt** | TCACTTCTGTCTC |
| **c+d-3nt** | TCACTTCTGTCT |
| **c+d-2nt** | TCACTTCTGTC |
| **Supplementary Figure S7. Choose the value of n, c and d** | |
| **n=8-Complementary** | BHQ1-GATCCTCAGCTCAGTAGAGTG ACCGAGA CAG ACCTCA GCATTAGC-BHQ1 |
| **n=8-Output_1_** | FAM-GCTAATGGTGAGGTCTG |
| **n=8-Output_2_** | CTACTGAGCAGAGGATC-HEX |
| **n=8-S** | GCTAATGCTGAGGTCTGTCTCGGT |
| **n=8-R** | GGTCACTCTACTGAGCTGAGGATC |
| **n=9-Complementary** | BHQ1-GATCCTCAGCTCAGTAGAGTGACCGAGA CAG ACCTCA GCATTAGC-BHQ1 |
| **n=9-Output_1_** | FAM-GCTAATGCAGAGGTCTG |
| **n=9-Output_2_** | CTACTGAGGTGAGGATC-HEX |
| **n=9-S** | GCTAATGCTGAGGTCTGTCTCGGT |
| **n=9-R** | GGTCACTCTACTGAGCTGAGGATC |
| **c3d4-Complementary** | BHQ1-GATCCTCAGCTCAGTAGATGACGCGAACAGACCTCAGCA  TTAGC-BHQ1 |
| **c3d4-Output_1_** | FAM-GCTAATGGTGAGGTCTG |
| **c3d4-Output_2_** | CTACTGAGCAGAGGATC-HEX |
| **c3d4-S** | GCTAATGCTGAGGTCTGTTCGCGT |
| **c3d4-R** | GCGTCATCTACTGAGCTGAGGATC |
| **c4d3-Complementary** | BHQ1-GATCCTCAGCTCAGTAGAGTGACCGAGACAGACCTCAGC  ATTAGC-BHQ1 |
| **c4d3-Output_1_** | FAM-GCTAATGCAGAGGTCTG |
| **c4d3-Output_2_** | CTACTGAGGTGAGGATC-HEX |
| **c4d3-S** | GCTAATGCTGAGGTCTGTCTCGGT |
| **c4d3-R** | GGTCACTCTACTGAGCTGAGGATC |
| **c5d2-Complementary** | BHQ1-GATCCTCAGCTCAGTAGAAAAA CCAAAAACAGACCTCA GCATTAGC-BHQ1 |
| **c5d2-Output_1_** | FAM-GCTAATGGTGAGGTCTG |
| **c5d2-Output_2_** | CTACTGAGCAGAGGATC-HEX |
| **c5d2-S** | GCTAATGCTGAGGTCTGTTTTTCC |
| **c5d2-R** | GTTTTTTCTACTGAGCTGAGGATC |
| **c6d1-Complementary** | BHQ1-GATCCTCAGCTCAGTAGAGGGGGAGGGGGACAGACCTC  AGCATTAGC-BHQ1 |
| **c6d1-Output_1_** | FAM-GCTAATGGTGAGGTCTG |
| **c6d1-Output_2_** | CTACTGAGCAGAGGATC-HEX |
| **c6d1-S** | GCTAATGCTGAGGTCTGTCCCCCT |
| **c6d1-R** | TCCCCCTCTACTGAGCTGAGGATC |

**Supplementary Table S2.** **Comparison of existing sequential logic circuits**

| **Study** | **Uniform Inputs** | **Reusable** | **Expandability in DNA circuits (Input and Output are DNA strands)** |
| --- | --- | --- | --- |
| **[1]** | **×** | **×** | **×** |
| **[2]** | **√** | **×** | **√** |
| **[3]** | **×** | **×** | **×** |
| **[4]** | **×** | **×** | **√** |
| **[5]** | **×** | **×** | **√** |
| **[6]** | **×** | **×** | **√** |
| **This** | **√** | **√** | **√** |

**Reference**

[1] C. Zhang, L. Shen, C. Liang, Y. Dong, J. Yang, J. Xu, *DNA Sequential Logic Gate Using Two-Ring DNA*, **2016**, *ACS Appl Mater Interfaces*, *8* (14), 9370, <https://doi.org/10.1021/acsami.6b00847>.

[2] W. Tang, W. Zhong, J. Fan, Y. Tan, Q. Huang, Y. Liu, *Addressable activated cascade DNA sequential logic circuit model for processing identical input molecules*, **2019**, *Chem Commun (Camb)*, *55* (45), 6381, <https://doi.org/10.1039/c9cc02632k>.

[3] J. Zhao, A. Pokhilko, O. Ebenhöh, S. J. Rosser, S. D. Colloms, *A single-input binary counting module based on serine integrase site-specific recombination*, **2019**, *Nucleic Acids Res*, *47* (9), 4896, <https://doi.org/10.1093/nar/gkz245>.

[4] D. Scalise, M. Rubanov, K. Miller, L. Potters, M. Noble, R. Schulman, *Programming the Sequential Release of DNA*, **2020**, *ACS Synth Biol*, *9* (4), 749, <https://doi.org/10.1021/acssynbio.9b00398>.

[5] Z. Guo, X. Zhang, S. H. Zhou, *Sequential logic circuit built on <i>λ</i> exonuclease for cross inhibition*, **2023**, *New Journal of Chemistry*, *47* (18), 8925, <https://doi.org/10.1039/d3nj00539a>.

[6] M. Zhang, C. Yancey, C. Zhang, J. Wang, Q. Ma, L. Yang, R. Schulman, D. Han, W. Tan, *A DNA circuit that records molecular events*, **2024**, *Sci Adv*, *10* (14), eadn3329, <https://doi.org/10.1126/sciadv.adn3329>.

**Supplementary Discussion**

**Supplementary Discussion S1. Detailed threshold calculations for the SR-latch consisting of NOR gates**

For the test results of the SR-latch consisting of NOR gates, when the 24th operation started, the average of the lowest fluorescence value when the output of the first 23 operations was 1 (22nd operation of the FAM channel and 20th operation of the HEX channel) and the highest fluorescence value when the output was 0 (8th operation of the FAM channel and 22nd operation of the HEX channel) was taken as the threshold value. Accordingly, following the completion of the reaction, if the fluorescence intensity surpasses 37 in the FAM channel, it indicates the 1 state of O_1_. Similarly, a fluorescence intensity exceeding 22 in the HEX channel signifies the 1 state of O_2_. The output_2_ of the HEX channel after the 24th operation is 1 in theory, but its fluorescence value is lower than 37 in the FAM channel, so output_2_ is actually 0 and the experiment was stopped after the 24th operation.
